# Role of Dust and Iron Solubility in Sulfate Formation during the Long-Range Transport in East Asia Evidenced by ^17^O-Excess Signatures

**DOI:** 10.1021/acs.est.2c03574

**Published:** 2022-09-15

**Authors:** Syuichi Itahashi, Shohei Hattori, Akinori Ito, Yasuhiro Sadanaga, Naohiro Yoshida, Atsushi Matsuki

**Affiliations:** † Sustainable System Research Laboratory (SSRL), Central Research Institute of Electric Power Industry (CRIEPI), Abiko, Chiba 270-1194, Japan; ‡ International Center for Isotope Effects Research (ICIER), Nanjing University, Nanjing 210023, Jiangsu, China; § School of Earth Sciences and Engineering, Nanjing University, Nanjing 210023, Jiangsu, China; ∥ Department of Chemical Science and Engineering, School of Materials and Chemical Technology, Tokyo Institute of Technology, Midori-ku, Yokohama 226-8502, Kanagawa, Japan; ⊥ Institute of Nature and Environment Technology, Kanazawa University, Kakuma-machi, Kanazawa 920-1192, Ishikawa, Japan; # Yokohama Institute for Earth Sciences, Japan Agency for Marine-Earth Science and Technology (JAMSTEC), Kanazawa-ku, Yokohama 236-0001, Kanagawa, Japan; ∇ Department of Applied Chemistry, Graduate School of Engineering, Osaka Metropolitan University, Naka-ku, Sakai 599-8531, Osaka, Japan; ○ Earth-Life Science Institute, Tokyo Institute of Technology, Meguro-ku, Tokyo 152-8551, Japan; ◆ National Institute of Information and Communications Technology, Koganei, Tokyo 184-8795, Japan

**Keywords:** sulfate aerosol, triple oxygen isotopes, downstream region, Asian dust, iron solubility

## Abstract

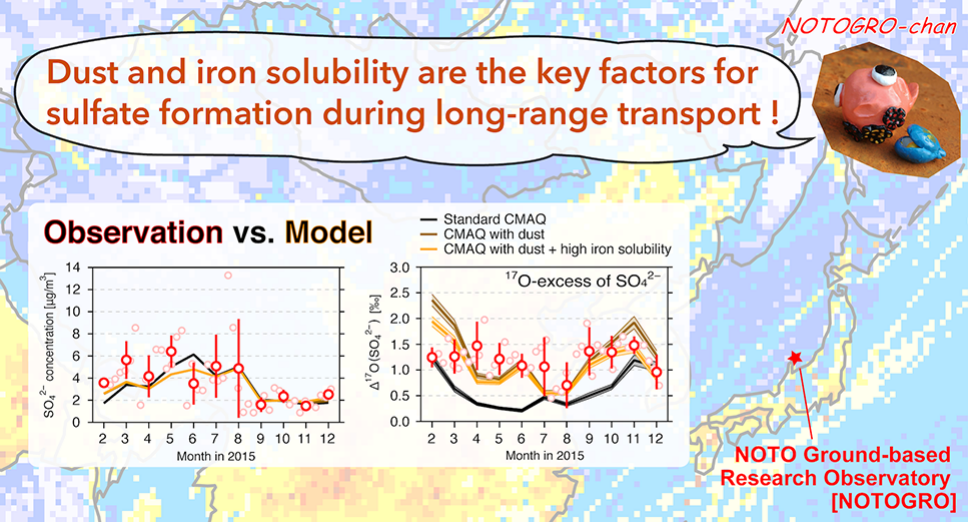

Numerical models have been developed to elucidate air pollution caused by sulfate aerosols (SO_4_^2–^). However, typical models generally underestimate SO_4_^2–^, and oxidation processes have not been validated. This study improves the modeling of SO_4_^2–^ formation processes using the mass-independent oxygen isotopic composition [^17^O-excess; Δ^17^O(SO_4_^2–^)], which reflects pathways from sulfur dioxide (SO_2_) to SO_4_^2–^, at the background site in Japan throughout 2015. The standard setting in the Community Multiscale Air Quality (CMAQ) model captured SO_4_^2–^ concentration, whereas Δ^17^O(SO_4_^2–^) was underestimated, suggesting that oxidation processes were not correctly represented. The dust inline calculation improved Δ^17^O(SO_4_^2–^) because dust-derived increases in cloud-water pH promoted acidity-driven SO_4_^2–^ production, but Δ^17^O(SO_4_^2–^) was still overestimated during winter as a result. Increasing solubilities of the transition-metal ions, such as iron, which are a highly uncertain modeling parameter, decreased the overestimated Δ^17^O(SO_4_^2–^) in winter. Thus, dust and high metal solubility are essential factors for SO_4_^2–^ formation in the region downstream of China. It was estimated that the remaining mismatch of Δ^17^O(SO_4_^2–^) between the observation and model can be explained by the proposed SO_4_^2–^ formation mechanisms in Chinese pollution. These accurately modeled SO_4_^2–^ formation mechanisms validated by Δ^17^O(SO_4_^2–^) will contribute to emission regulation strategies required for better air quality and precise climate change predictions over East Asia.

## Abstract 2

Dust and high iron solubilities are identified as important factors for SO_4_^2−^ formation in the long-range transport over East Asia.

## 1 Introduction

The formation of sulfate aerosols (SO_4_^2–^) in East Asia is a concern because it causes severe haze and pollution events, particularly in China, resulting in low visibility^1^ and public health problems.^2^ SO_4_^2–^ is also one of the important short-lived climate forcers (SLCFs) related to regional and global climate changes.^3,4^ Atmospheric SO_4_^2–^ is produced mainly via the oxidation of sulfur dioxide (SO_2_),^5^ and SO_2_ emissions from China accounted for approximately a quarter of global total emissions.^6^ The problem of SO_4_^2–^ pollution in East Asia is not confined to the emission sources in China because of the relatively long lifetime of SO_4_^2–^ and the westerlies in the mid-latitudes.^7^ Thus, the long-range transport of SO_4_^2–^ from the source region on the Asian continent to the downstream region of the air masses (i.e., Republic of Korea and Japan) has been thoroughly investigated.^8−10^

To elucidate pollution caused by atmospheric SO_4_^2–^, chemical transport models (CTMs) have been developed and used in various studies. In CTMs, the gas-phase oxidation by the hydroxyl (OH) radical and the aqueous-phase oxidations by hydrogen peroxide (H_2_O_2_), ozone (O_3_), and O_2_ catalyzed by transition-metal ions (TMIs) for SO_4_^2–^ formation are typically considered. However, typical CTMs often underestimate the burden of atmospheric SO_4_^2–^, particularly in China,^11,12^ suggesting that some SO_4_^2–^ formation in the atmosphere is missing. To date, heterogeneous SO_4_^2–^ production mechanisms particularly for aerosol surfaces^13−16^ have been proposed to explain this missing formation such as the enhanced role of the oxidation by reactive nitrogen,^17−20^ H_2_O_2_,^21^ TMI-catalyzed O_2_,^22−24^ and Mn-catalyzed oxidation.^25,26^ Nonetheless, the observational evidence has not yet identified a specific mechanism. For example, while the importance of NO_2_ for SO_4_^2–^ formation within the aerosol surface was proposed,^17^ follow-up studies have cast doubt on the impact of its reaction.^25,26^ Thus, these proposed reactions have been highly controversial arguments.

These pollution and haze events over China and their impact on the downstream region should be mitigated, mainly via the reduction of anthropogenic SO_2_ emissions. However, according to reports in Western countries, atmospheric SO_4_^2–^ has declined less rapidly than would be expected from decreases in SO_2_ emissions.^27,28^ This unknown response has been attributed to chemical feedback mechanisms of a weakening H_2_O_2_ limitation on the S(IV) + H_2_O_2_ pathway^29^ and acidity-driven enhancement of the S(IV) + O_3_ pathway under low SO_2_ conditions^30,31^ in Western countries. Therefore, accurate CTMs for both concentration and oxidation processes are required to establish an effective emission regulation strategy for improving air quality, and the accurate implementations of atmospheric SO_4_^2–^ formation mechanisms in CTMs are essential for elucidating present pollution and predicting future air quality. Yet, the atmospheric SO_4_^2–^ formation pathways implemented in CTMs have been simply evaluated by total SO_4_^2–^ masses and not validated by independent observational evidence.

One proven method to validate atmospheric SO_4_^2–^ formation is the mass-independent oxygen isotopic composition (Δ^17^O)^32^ of SO_4_^2–^, which reflects the formation pathway from SO_2_ to SO_4_^2–^. The comparison of observed and modeled Δ^17^O(SO_4_^2–^) has enabled the recognition and quantification of SO_4_^2–^ formation mechanisms, including TMI-catalyzed S(IV) + O_2_,^33^ SO_4_^2–^ formation by O_3_ oxidation on sea salt aerosol,^34^ S(IV) oxidation by hypohalous acids,^35,36^ and acidity-driven changes in SO_4_^2–^ formation pathways.^31,37^ For extreme pollution events in China, the importance of the heterogeneous chemistry of SO_4_^2–^ production, which is not considered in typical CTMs, has been discussed based on Δ^17^O(SO_4_^2–^);^13,14^ however, verification has been limited in China. Studies to reveal the SO_4_^2–^ formation process in the long-range transport over East Asia using Δ^17^O(SO_4_^2–^) have been limited.

**Figure 1:**
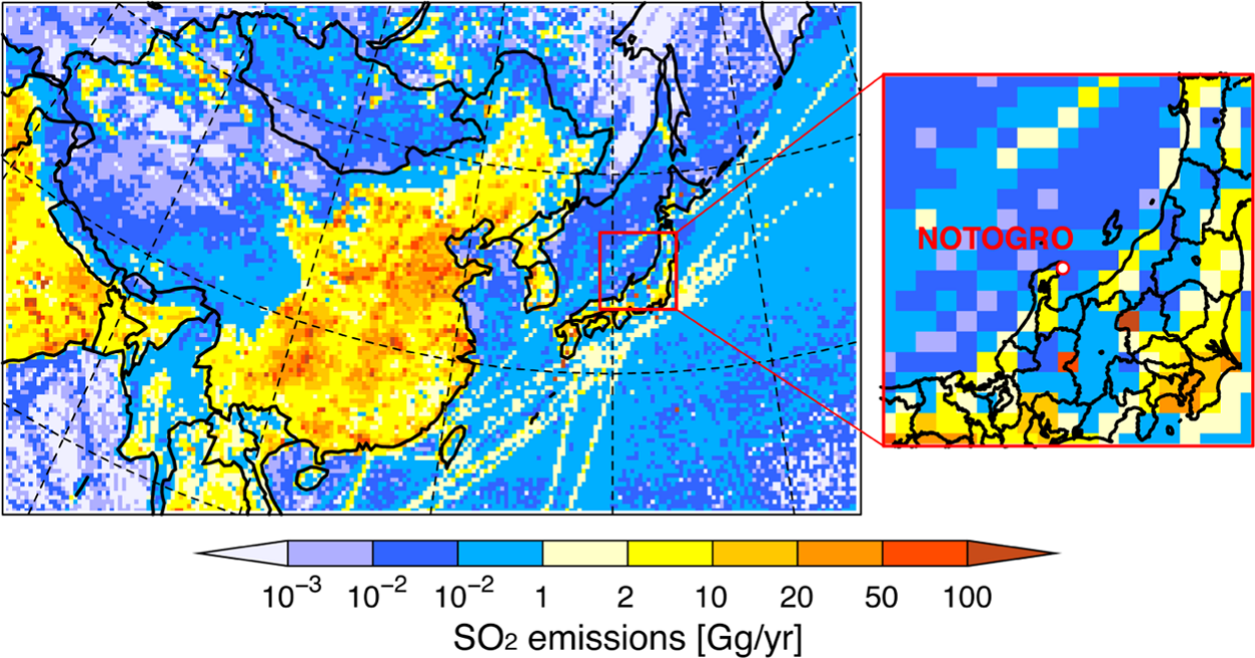
SO_2_ emissions compiled in this study plotted over the modeling domain, and the location of the NOTOGRO observation site.

SO_2_ emissions in East Asia have recently begun to decrease.^38^ Without the validation of SO_4_^2–^ formation processes in East Asia, studies using current typical CTMs may not adequately predict air quality and climate change, given the possible cause of chemical feedback mechanisms, which have been studied in Western countries.^27,28^ Therefore, the observation of Δ^17^O(SO_4_^2–^) in regions downstream from intense emission sources, such as China, is critical for research into SO_4_^2–^. The present study describes the annual observation of SO_4_^2–^ and Δ^17^O(SO_4_^2–^) in 2015 at the background site in Japan (NOTO Ground-based Research Observatory [NOTOGRO] located over the downstream region of China) (Figure 1). Regional CTM analyses over East Asia are conducted with the Community Multiscale Air Quality (CMAQ) model with three different configurations (Exps. A, B, and C). The role of mineral dust as a supply of alkaline material,^20,39,40^ which is a unique aspect of the East Asian environment, is investigated because dust-driven higher pH alters acidity-dependent aqueous-phase SO_4_^2–^ formation, such as TMI-catalyzed oxidation by O_2_ and oxidation by O_3_. In addition, the impact of TMIs on SO_4_^2–^ formation is examined because these are uncertain parameters for emissions and solubilities in the model.^41^ Through comparisons of observed and modeled SO_4_^2–^ and Δ^17^O(SO_4_^2–^) at NOTOGRO in Japan, this study clarifies the key factors controlling the formation process of long-range-transported SO_4_^2–^ in East Asia.

## 2 Methods

### 2.1 Observation of Aerosol and Oxygen Isotopic Composition in Japan

The atmospheric observations were performed at NOTOGRO at 37.45°N, 137.36°E (Figure 1). The Noto peninsula extends from the west coast of mainland Japan approximately 150 km into the sea of Japan, and NOTOGRO is located on the tip of this peninsula. The geographical location of NOTOGRO is ideal for capturing the atmospheric variation in East Asia because it is surrounded by the sea and isolated from major pollution sources in Japan.^42^

The aerosol samples were collected by a high-volume air sampler (MODEL-120 SL, Kimoto Co., Ltd., Japan) mounted on the rooftop (∼10 m above sea level) of NOTOGRO. Fine (<2.5 μm) and coarse (>2.5 μm) samples were collected on prebaked (450 °C for 4 h) quartz filters (2500QAT-UP, Pall Co., Ltd.; TE-230-QZ, Tisch Environmental Inc.). Sampling was performed at a flow rate of ∼1.05 m^3^/min, and the sampling interval was usually 1 or 2 weeks. After sampling, the filters were wrapped in aluminum foil, sealed in polyethylene bags, and stored in a clean freezer at −20 °C prior to the measurement at the Tokyo Institute of Technology, Japan. In the laboratory, half of each filter was soaked in ultrapure water (30 mL) in a 50 mL centrifuge tube (Centricon plus-70, Millipore). The sample solution was separated from the insoluble materials and the filter by centrifuging in a centrifugal filter unit for 10 min. This method can recover >98% of the initial water volume. The major anions were quantified using an ion chromatograph (Dionex ICS-2100, Thermo Fisher Scientific) with a guard column (Dionex IonPac AG19, Thermo Fisher Scientific) and a separation column (Dionex IonPac AS19, Thermo Fisher Scientific). The major cations were quantified using an ion chromatograph (881 Compact IC Pro, Metrohm, Switzerland) with a guard column (Metrosep C4 S-Guard/4.0, Metrohm) and a separation column (Metrosep C4-150/4.0, Metrohm). The uncertainties of measurement errors were estimated by analyzing five different concentration standards at intervals of every ∼20 sample measurements, resulting in approximately 4% for both cation and anion measurements.

The measurement procedures for Δ^17^O(SO_4_^2–^) are described in our previous studies.^31,37,43^ Briefly, 1 or 2 μmol of H_2_SO_4_ separated by ion chromatography was chemically converted to Na_2_SO_4_, and 30% of H_2_O_2_ solution (1 mL) was added, and the mixture was dried. The Na_2_SO_4_ was converted to silver sulfate (Ag_2_SO_4_) using an ion-exchange resin. This Ag_2_SO_4_ powder was transported in a custom-made quartz cup, which was dropped into the furnace of a high-temperature conversion elemental analyzer (TC/EA, Thermo Fisher Scientific) at 1000 °C and thermally decomposed into O_2_. The O_2_ gas was introduced separately into an isotope ratio mass spectrometer to measure *m*/*z* = 32, 33, and 34. The Δ^17^O(SO_4_^2–^) measurements were corrected using our working standard B (Δ^17^O(SO_4_^2–^) = 2.4‰) with the same procedure described previously.^31,37,43^ In this correction for isotopic analysis, SD (1σ) for the corrected values for standard B was 0.11‰ based on measurements for the samples collected in 2015, and this 1σ uncertainty was used for the error of the isotopic measurements in the present study. The raw observation data are presented in the Supporting Information (Table S1). All data for SO_4_^2–^ concentration and Δ^17^O(SO_4_^2–^) are corrected using Na^+^ concentration to non-sea salt fraction of SO_4_^2–^ (nss-SO_4_^2–^) in a similar manner reported previously.^31^

### 2.2 Regional Chemical Transport Modeling over East Asia

#### 2.2.1 Model Description

The regional air quality modeling was conducted with the CMAQ model version 5.3.1.^44,45^ In this study, the simulation domain covered the entirety of East Asia with a horizontal resolution of 36 km (Figure 1) and 44 nonuniform layers from the surface to 50 hPa. One gas-phase reaction and five aqueous-phase reactions in cloud are involved in SO_2_ oxidation (i.e., SO_4_^2–^ formation) in the original CMAQ. The one gas-phase reaction is SO_2_ oxidation by OH (GAS), and the five oxidants in the aqueous-phase reactions in cloud are H_2_O_2_ (AQ(H_2_O_2_)), O_3_ (AQ(O_3_)), O_2_ catalyzed by TMIs (AQ(O_2_)), peroxyacetic acid (PAA) (AQ(PAA)), and methyl hydrogen peroxide (MHP) (AQ(MHP)). In addition to these five oxidants in the original CMAQ, taking into account the elevated NO_2_ concentration in Chinese pollutions,^17−19^ the aqueous-phase pathway in cloud via NO_2_ was added in this study. This inclusion partly improved the model underestimation issue during winter in our previous study.^46^ Note that the production of SO_4_^2–^ on the aerosol surface is not considered in this study because the specific Chinese haze events that occurred in limited areas are out of scope and our focus is on capturing SO_4_^2–^ pollution over East Asia from the viewpoint of the background site in Japan.

This version 5.3.1 of CMAQ has several features to achieve the purpose of this study. At first, Fe and Mn emissions can be treated as independent variables, although these emissions had been previously dependent on the total PM_2.5_ emission. Thus, we implemented the Transition Metal Inventory-Asia version 1.0^47^ to calculate the emissions of Fe and Mn. Furthermore, although the default setting of CMAQ does not possess, we implemented the pH dependency rate constant for the calculation of AQ(O_2_) using the synergistic relationship between Fe and Mn developed in our previous study.^46^ These explicit treatments of Fe and Mn concentrations are major advantages of CMAQ and our study because other CTMs, such as GEOS-Chem, treat TMI concentrations as a fraction of the PM_2.5_ concentration. Because of these improvements for Fe and Mn emissions and the rate constant for the calculation for AQ(O_2_), we tested the remaining uncertainty of solubilities of Fe and Mn, as described in Section 2.2.2.

Second, it is possible to implement the developed physics-based inline dust calculation after CMAQ version 5.2. Before this version, the effect of soil dust for neutralization and altering cloud drop pH could not be considered, even though one of the characteristics in East Asia is the role of mineral dust originating from the Taklamakan and Gobi Deserts, the Loess Plateau, and Inner Mongolia. However, dust is not implemented as the default setting of CMAQ, which hampers precise calculation for the pH-dependent SO_4_^2–^ formation pathways, especially for AQ(O_3_) and AQ(O_2_). The impact on SO_4_^2–^ formation caused by the difference with or without implementation of inline dust calculation was tested in this study, as explained in Section 2.2.2. The details of the modeling description and chemical configurations are, respectively, given in the Supporting Information (Sections S1 and S2).

#### 2.2.2 Model Experiments

In this study, we compared the following three experiments (Exp. A, Exp. B, and Exp. C) to investigate the role of mineral dust and solubilities of Fe and Mn for SO_4_^2–^ formation, as summarized in Table 1. In Exp. A, which is the original settings in the CMAQ model version 5.3.1, the solubilities of anthropogenic Fe and Mn were set as 10 and 50% and dust inline calculation was not implemented (Table 1).

**Table 1:** Modeling Experimental Design and Settings of Solubility for Anthropogenic and Dust TMIs^a^

		Fe		Mn	
modeling experiments	design	anthropogenic (%)	dust (%)	anthropogenic (%)	dust (%)
Exp. A	the standard simulation	10	-	50	-
Exp. B	incorporation of dust inline calculation	10	1	50	50
Exp. C	same as Exp. B, but increasing TMI solubilities	54	3	97	50

a Note: dash means dust simulation was not implemented in Exp. A. In the aqueous-phase reaction of O_2_ catalyzed by TMIs (AQ(O_2_)), Fe(III), and Mn(II) are related. Fe(III) was assumed to be 10% of the total dissolved Fe during the day and 90% at night as the diurnal variation. Mn(II) was assumed to be the same for all dissolved Mn. These diurnal variations of Fe(III) and Mn(II) were the same in all three experiments.

In Exp. B, the modulation of pH by mineral dust was considered, and thus the newly developed dust inline calculation scheme in CMAQ^48^ was applied. In addition to pH changes by mineral dust, the dust-derived Fe and Mn were considered in Exp. B. The solubility of dust Fe and Mn was set as 1 and 50%, respectively^33^ (Table 1). The details of the dust inline calculation are given in the Supporting Information (Section S3).

In Exp. C, increased TMI solubilities were considered because one of the uncertainties in modeling settings still under debate is TMI solubilities. Based on a literature review, the range of TMI solubility for Fe and Mn are 0.03–54 and 1.2–97%, respectively.^14^ The solubilities of anthropogenic Fe and Mn were set at 54 and 97%, respectively, for the maximum possible production through TMI processes (Table 1). Based on the integrated massively parallel atmospheric chemical transport (IMPACT) global aerosol model^41^ (Figures S1 and S2 in the Supporting Information), the solubility of dust Fe was increased from 1% in Exp. B to 3% in Exp. C (Table 1). The result from the IMPACT global aerosol model also suggested the verification of increasing anthropogenic Fe solubility (Figures S3 and S4 in the Supporting Information). The discussion of TMI solubilities is also given in the Supporting Information (Section S4).

#### 2.2.3 Calculation of Δ^17^O(SO_4_^2–^) from Model Outputs

The diagnostic tool of the sulfur tracking method in CMAQ was used to output each oxidation process involved in SO_4_^2–^ formation. From these outputs in CMAQ, the modeled Δ^17^O(SO_4_^2–^) was derived based on eqs 1 and 2.
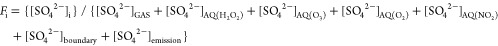
1
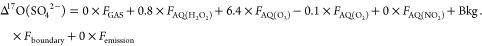
2In these equations, [SO_4_^2–^]_i_ is the SO_4_^2–^ concentration from each process and *F*_i_ represents the fractional contribution for each process, where i indicates the SO_4_^2–^ formation process. The contributions of PAA and MHP were negligible (around 0.1% contribution throughout the year) and hence were omitted from this calculation. In this study, Δ^17^O(SO_4_^2–^) of 0‰ was set for the GAS and emission oxidation pathways,^49^ and the end members and their uncertainties for Δ^17^O(SO_4_^2–^) in the other oxidation pathways were set to 0.8 ± 0.2‰ for AQ(H_2_O_2_), 6.4 ± 0.3‰ for AQ(O_3_), and −0.1‰ for AQ(O_2_) (see our previous studies^31,37,43^ for the determination of the end members). The value of AQ(NO_2_) was set as 0‰ according to He et al.^13^ and references therein. Δ^17^O(SO_4_^2–^) = 0‰ for SO_4_^2–^ produced by AQ(NO_2_) is expected based on the following three mechanisms: radical chain mechanism,^50^ oxygen-atom transfer from OH^–^,^51^ or from O_2_.^52^ Additionally, a detailed discussion of the uncertainties of the AQ(O_3_) end member is given in the Supporting Information (Section S5 and Figure S5). The SO_4_^2–^ derived from the boundary conditions is considered the background existing SO_4_^2–^, and thus monthly values observed at Alert, Canada, ranging from 0.5 to 1.3‰^33^ were used.

#### 2.2.4 Statistical Analysis

The model performance for SO_4_^2–^ concentration and calculated Δ^17^O(SO_4_^2–^) was statistically evaluated. The metrics were correlation coefficient (*R*), normalized mean bias (NMB), and normalized mean error (NME).
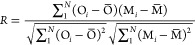
3
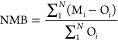
4
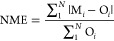
5Here, *N* is the total number of paired observations (O) and models (M), and these averages are denoted as O̅ and M̅, respectively. The recommended metrics for SO_4_^2–^ concentration are model performance goals for best performance of *R* > 0.7, NMB < ±10%, and NME < +35% and model performance criteria for acceptable performance of *R* > 0.4, NMB < ±30%, and NME < +50%.^53^

## 3 Results and Discussion

### 3.1 Observed and Simulated SO_4_^2–^ Characteristics over East Asia

**Figure 2:**
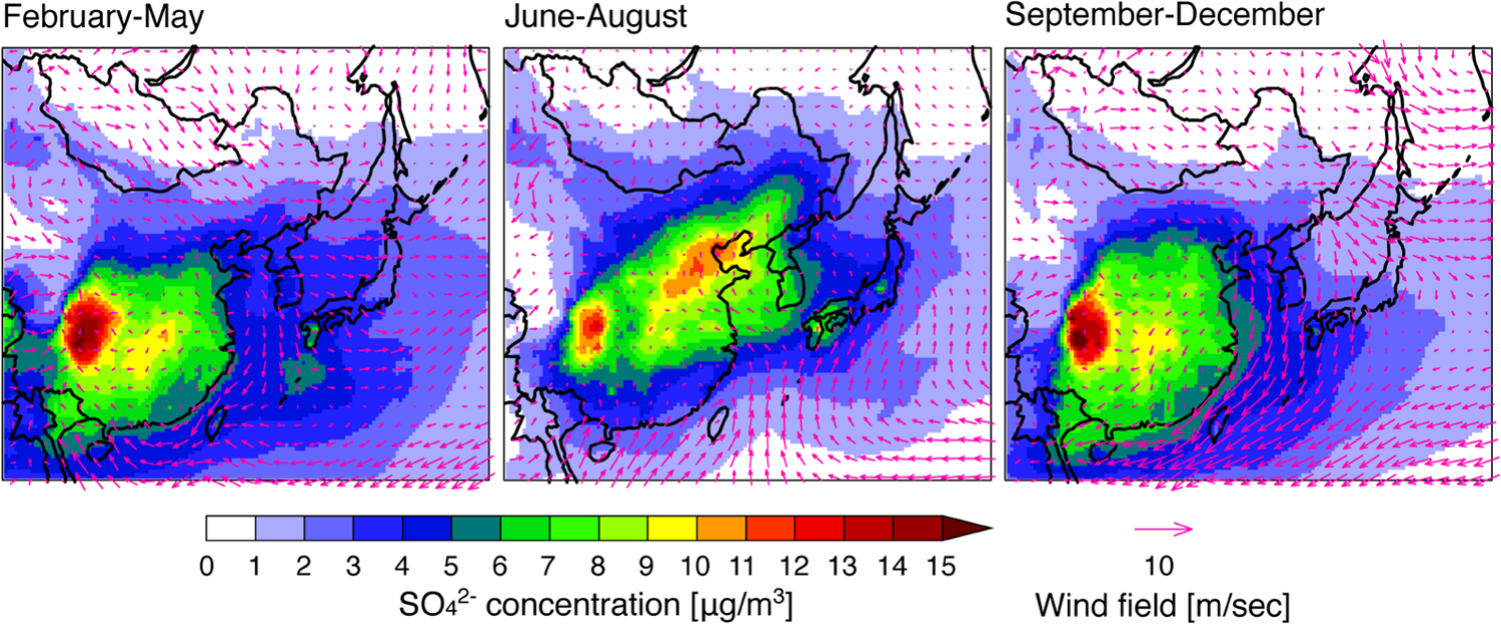
Spatial distribution of simulated SO_4_^2–^ with wind field over East Asia during late winter to spring (February–May), summer (June–August), and autumn to winter (September–December).

**Figure 3:**
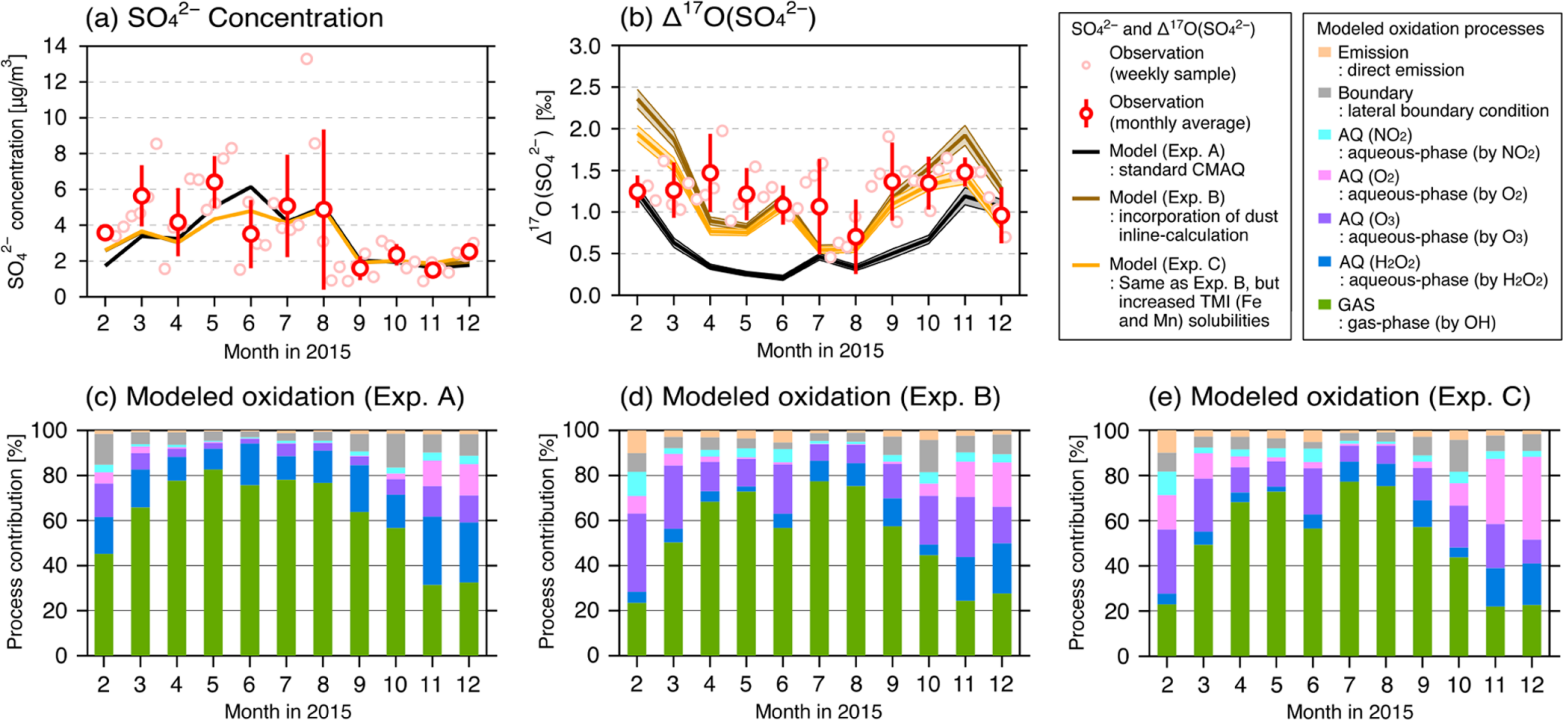
Observed and simulated (a) SO_4_^2–^ concentration and (b) Δ^17^O(SO_4_^2–^) from February to December 2015. Simulated SO_4_^2–^ formation in (c) Exp. A, (d) Exp. B, and (e) Exp. C. Each oxidation process is normalized to the simulated SO_4_^2–^ concentration and is shown as relative percentages. The values for this figure are listed in the Supporting Information (Tables S2–S4).

The simulated spatial distribution of SO_4_^2–^ over East Asia was divided into three seasons, late winter to spring (February–May), summer (June–August), and autumn to winter (September–December), to characterize seasonal behaviors (Figure 2). The highest SO_4_^2–^ concentrations were found over mainland China, and the higher-concentration regions extended into the downstream region over the Korean Peninsula and Japan. This feature was dominant from spring to summer; therefore, the observations in the downstream region of Japan detected the polluted air mass resulting from the long-range transport over East Asia. In contrast, from autumn to winter when the strong northwesterly wind field by the Asian monsoon was dominated, the SO_4_^2–^ concentration was low and was characterized by clean background conditions. The weekly and monthly average variations of SO_4_^2–^ concentration observed at NOTOGRO also showed higher concentrations from spring to summer and lower concentrations during autumn to winter (Figure 3a). As the background site in Japan, Chichijima island located in the western North Pacific (i.e., south of Tokyo) showed higher concentration in winter and lower concentration in summer.^54^ The difference in the seasonal variation of SO_4_^2–^ concentration seen in NOTGRO is caused by the outflow pattern in East Asia, as found in Figure 2. Overall, throughout the year, the statistical analyses showed that all three modeling experiments (Exps. A, B, and C) generally captured the SO_4_^2–^ concentration (Table 2).

**Table 2:** Statistical Analysis of Model Performance for the Monthly Average of SO_4_^2–^ and the Monthly Weighted Average of Δ^17^O(SO_4_^2–^)^a^

components	SO_4_^2–^			Δ^17^O(SO_4_^2–^)		
metrics	Exp. A	Exp. B	Exp. C	Exp. A	Exp. B	Exp. C
mean (obs.)	3.80			1.20		
mean (model)	3.43	3.26	3.30	0.57	1.21	1.03
*R*	0.67	**0.79**	**0.79**	0.23	**0.47**	**0.48**
NMB (%)	–12.42	–15.08	–13.86	–47.70	**+7.13**	**–9.69**
NME (%)	+28.63	**+24.34**	**+23.59**	+49.24	**+34.38**	**+24.95**

a Note: units of mean are μg/m^3^ for SO_4_^2–^ and ‰ for Δ^17^O(SO_4_^2–^), respectively. The score improvement compared with Exp. A is shown in bold font.

The weekly observed Δ^17^O(SO_4_^2–^) at NOTOGRO ranged from 0.46 to 1.98‰, and the monthly weighted average ranged from 1.0 to 1.5‰, except for a value of 0.70‰ in August (Figure 3b). These observed Δ^17^O(SO_4_^2–^) values ranging from 1.0 to 1.5‰ were higher than those reported in the polluted region in China, where the observations from Beijing varied from 0.1 to 1.6‰ with a mean of 0.9 ± 0.3‰^13^ and the data from Wuhan varied from 0.14 to 1.02‰ with a time-weighted average of 0.53‰.^23^ The observed higher Δ^17^O(SO_4_^2–^) values at NOTOGRO are explained by either the greater importance of AQ(O_3_) (Δ^17^O(SO_4_^2–^) = 6.4 ± 0.3‰) or the lesser importance of pathways such as GAS (Δ^17^O(SO_4_^2–^) = 0‰) and AQ(O_2_) (Δ^17^O(SO_4_^2–^) = −0.1‰). We note that the low Δ^17^O(SO_4_^2–^) values with high SO_4_^2–^ concentration in August probably originated from volcanic eruptions in western Japan, which is discussed in Section 3.2.

### 3.2 Disagreement between Observed and Modeled Δ^17^O in the Standard Model

In the standard CMAQ model experiment (Exp. A), although the modeled SO_4_^2–^ concentrations agreed with the observations (Figure 3a, black line), the modeled Δ^17^O(SO_4_^2–^) ranged from 0.21 to 1.24‰ and underestimated the observations over the period except in winter (Figure 3b, black line). In Exp. A, GAS was the dominant oxidation process, contributing more than 30% of SO_4_^2–^ formation during the period and reaching up to 80% during spring and summer. Following the GAS process, AQ(H_2_O_2_) was the next most important process, contributing 10–30% of SO_4_^2–^ formation (Figure 3c). In the model, a higher SO_4_^2–^ concentration with lower Δ^17^O(SO_4_^2–^) was found in August, and the low Δ^17^O(SO_4_^2–^) was due mainly to the high contribution of the GAS process (Δ^17^O(SO_4_^2–^) = 0‰). The importance of GAS in volcano-originated SO_4_^2–^ was suggested in this study, whereas the importance of AQ(O_2_) was suggested to be the dominant process in volcanic plumes.^55^ This difference indicates that the oxidation processes contributing to SO_4_^2–^ formation inside a plume are different from those contributing to SO_2_ oxidation after diffusion into the atmosphere. The discussion of this volcanic impact is given in the Supporting Information (Section S6 and Figure S6). Overall, the underestimation of Δ^17^O(SO_4_^2–^) in Exp. A indicates that the standard model parameterization in CMAQ is missing oxidation pathways that increase Δ^17^O(SO_4_^2–^).

### 3.3 Importance of Dust-Derived pH Increase in SO_4_^2–^ Formation

Given the importance of dust in SO_4_^2–^ formation and neutralization,^22,37,56^ the dust inline calculation was implemented in Exp. B. The supply of alkaline dust to the atmosphere increases the pH and thus increases the acidity-dependent reaction rates for AQ(O_2_) and AQ(O_3_).^5^ In Exp. B, the dust-derived Fe and Mn were calculated in addition to anthropogenic Fe and Mn. Although Exp. B did not improve the estimation of SO_4_^2–^ concentration substantially compared with Exp. A (Figure 3a and Table 2), Δ^17^O(SO_4_^2–^) for Exp. B ranged from 0.56 to 2.36‰ (Figure 3b, brown line; Table 2). For the relative contributions of the SO_2_ oxidation processes in the model, AQ(O_3_) increased, whereas GAS decreased (Figure 3d). This switch can be explained by the neutralization of atmospheric acidity by dust-derived CaCO_3_, and the increased pH obtained by including the inline dust calculation is consistent with previous works.^22,40^ The results in Exp. B showed better agreement with the observations of Δ^17^O(SO_4_^2–^) (Table 2), and this improvement in Δ^17^O(SO_4_^2–^) in Exp. B suggests that mineral dust supply is crucial in increasing the acidity-driven SO_4_^2–^ formation in East Asia. However, the overestimation of Δ^17^O(SO_4_^2–^) from February to March and from October to December remained.

### 3.4 Importance of TMI Solubilities in SO_4_^2–^ Formation

To account for the remaining gap between the model and observed values of Δ^17^O(SO_4_^2–^), the increases in TMI solubilities in the polluted air were considered in Exp. C. In the standard model of Exp. A, although the anthropogenic emission of TMIs is accurately considered based on the latest emission data set, the modeled estimates of solubilities of anthropogenic and dust Fe and Mn remain highly uncertain in cloud water, as pointed out previously.^14,29,31,33^ The increased Fe solubilities have been indicated based on global model comparisons with multiple field campaigns over the Northwest Pacific^57^ and also observed toward the downstream region of East Asia,^58,59^ mainly because fine mineral aerosols can be acidified due to air pollution. Thus, for Exp. C, we implemented the higher solubilities for both anthropogenic and dust Fe. Compared to the anthropogenic Fe solubility (10%) and Mn solubility (50%) considered in Exps. A and B, the maximum solubility for anthropogenic Fe (54%) and Mn (97%) over the literature^14^ was taken in Exp. C (Table 1). Note that the higher solubility of Fe in dust (3%) considered in Exp. C is generally consistent with that simulated by the IMPACT global aerosol model^57^ (Figures S1 and S2 in the Supporting Information).

The SO_4_^2–^ concentration in Exp. C did not increase substantially compared with that in Exps. A and B (Figure 3a and Table 2), but the overestimation in modeled Δ^17^O(SO_4_^2–^) from February to March was clearly decreased in Exp. C (Figure 3b, orange line; Table 2) and ranged from 0.54 to 1.94‰. The improvement in Δ^17^O(SO_4_^2–^) values was explained by the increased contribution of AQ(O_2_) owing to the higher solubilities of Fe and Mn (Figure 3e), especially from October to December. Regarding the importance of the AQ(O_2_) process, TMI concentration levels have been discussed in China. In Beijing, TMI-catalyzed oxidation showed a clear distinguishment of higher/lower contribution during polluted/clean periods.^14^ In Wuhan, the enhanced role of TMI-catalyzed oxidation in winter was suggested due to higher PM_2.5_ concentration.^23^ In contrast, this study highlights the role of TMI-catalyzed AQ(O_2_) through the consideration of solubilities over the downstream region of East Asia, as evidenced by Δ^17^O(SO_4_^2–^). The importance of TMI solubilities, which led to the improvement of Δ^17^O(SO_4_^2–^), was found during February and March when SO_4_^2–^ concentration was higher and from October to December when SO_4_^2–^ concentration was lower. Therefore, it is concluded that the role of TMI-catalyzed AQ(O_2_) by enhancing solubilities does not depend on the pollution level in this case. Overall, the increased pH obtained by including dust and the increase in TMI solubilities in Exp. C showed the best match for SO_4_^2–^ concentration and Δ^17^O(SO_4_^2–^) among three experiments conducted in this study. The series of results strongly indicate the importance of dust and TMI solubilities for SO_4_^2–^ formation via changes in oxidation processes in downstream regions of East Asia, which was not discovered in previous studies that considered only SO_4_^2–^ mass.

### 3.5 Toward Closer Agreement between Observed and Modeled SO_4_^2–^ Formation

However, SO_4_^2–^ concentration was still underestimated from February to March (observed values: 3.58 ± 0.26 and 5.64 ± 1.71 μg/m^3^; modeled values in Exp. C: 2.61 and 3.66 μg/m^3^; Table S2 in the Supporting Information) and Δ^17^O(SO_4_^2–^) values were overestimated, even in Exp. C (observed values: 1.24 ± 0.20 and 1.26 ± 0.33‰; modeled values in Exp. C: 1.94 ± 0.09 and 1.57 ± 0.08‰; Table S3 in the Supporting Information). Since the domestic contribution to SO_4_^2–^ in Japan is estimated to be small except in summer,^8,9^ the most plausible reason for missing processes is attributed to inadequate SO_4_^2–^ formation in polluted areas over China. Here, Δ^17^O(SO_4_^2–^) values of possible missing processes (defined as Δ^17^O(SO_4_^2–^)_missing_) are calculated from the following mass-balance calculation
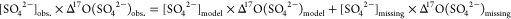
6where [SO_4_^2–^]_obs._, [SO_4_^2–^]_model_, and [SO_4_^2–^]_missing_ are SO_4_^2–^ concentrations for observation, model, and missing processes (i.e., difference between the observation and model), respectively, and Δ^17^O(SO_4_^2–^)_obs._, Δ^17^O(SO_4_^2–^)_model_, and Δ^17^O(SO_4_^2–^)_missing_ are Δ^17^O(SO_4_^2–^) for the observation, model, and missing processes, respectively. Given that [SO_4_^2–^]_missing_ is calculated by [SO_4_^2–^]_obs._ minus [SO_4_^2–^]_model_, Δ^17^O(SO_4_^2–^)_missing_ can be obtained from eq 6. The calculated Δ^17^O(SO_4_^2–^)_missing_ was −0.63 ± 0.52‰ during February and 0.70 ± 1.01‰ during March, respectively.

For the calculated Δ^17^O(SO_4_^2–^)_missing_ of −0.63 ± 0.52‰ during February, the result can be explained by SO_4_^2–^ formation via TMI-catalyzed oxidation by O_2_, Mn-catalyzed oxidation, and other reactions (i.e., oxidation by NO_2_) having Δ^17^O(SO_4_^2–^) close to 0‰. This interpretation is consistent with previous studies, which proposed these mechanisms as missing processes in Chinese haze.^17,18,24−26^ It is worthy to conclude that AQ(H_2_O_2_) does not explain this missing SO_4_^2–^ formation during February.

Conversely, for the calculated Δ^17^O(SO_4_^2–^)_missing_ of 0.70 ± 1.01‰ during March, this value is close to Δ^17^O(SO_4_^2–^) = 0.8 ± 0.2‰ for AQ(H_2_O_2_), although we could not fully exclude the possibilities of other reactions that have lower Δ^17^O(SO_4_^2–^). In terms of the SO_4_^2–^ formation process, faster H_2_O_2_ oxidation of SO_4_^2–^ formation in high solute strength was suggested in Chinese haze events.^21^ The current CTMs are based on kinetics research in dilute aqueous solutions and may miss such strengthened features in the atmosphere. The contribution of AQ(H_2_O_2_) was declined in Exp. C compared to that in Exp. A (Figure 3e,c) through increased dust-derived pH and enhanced TMI solubilities in this study. Because AQ(H_2_O_2_) does not depend on pH, faster oxidation by H_2_O_2_ in Chinese pollution would also affect the downstream region over East Asia even in the application of Exp. C. In this study, although we do not include reactions on the aerosol surface because our focus is not on the Chinese haze itself, the accurate modeling to capture the enhanced SO_4_^2–^ concentration in haze events will also improve SO_4_^2–^ behaviors in the downstream region if the long-range transport occurs.

To date, SO_4_^2–^ production mechanisms have been proposed to explain this missing formation in Chinese pollution and haze events, as introduced. Although this study cannot identify a single mechanism for this missing formation pathway, our results imply that the oxidation pathways for inadequate SO_4_^2–^ formation in polluted areas over China are not always identical. Based on our Δ^17^O(SO_4_^2–^) approach, these estimations for missing processes from the perspective of the downstream region in East Asia are the first supportable information for unexplained SO_4_^2–^ formation over China. A recent study^26^ proposed the dominance of Mn-catalyzed oxidation of SO_2_ and negligible formation pathways by gas- and aqueous-phase reactions in Chinese haze because the meteorological condition of the stable boundary layer with weak turbulence prohibits their formations. Under such conditions dominated by the Mn-catalyzed oxidation pathway, the value of Δ^17^O(SO_4_^2–^) is expected to be close to 0‰; however, the reported value of Δ^17^O(SO_4_^2–^) in Beijing haze from October 2014 to January 2015 ranged from 0.1 to 1.6‰ with a mean of 0.9 ± 0.3‰.^13^ Therefore, the dominance of Mn-catalyzed oxidation and the negligible contributions from other processes will be required to be carefully examined in terms of the validation with Δ^17^O(SO_4_^2–^) in the future study. To validate SO_4_^2–^ formation over East Asia covering from the haze above intense emission sources to a background condition over the downstream region, furthermore studies including laboratory experiments, measurements of both SO_4_^2–^ concentration and Δ^17^O(SO_4_^2–^), and numerical modeling are needed.

### 3.6 Implications for Future Air Quality and Climate Studies

We demonstrated the effect of the dust-derived increase in pH on SO_4_^2–^ production and the need to increase TMI solubilities in the modeling by constraining both SO_4_^2–^ concentration and Δ^17^O(SO_4_^2–^) at the background site in Japan over the downstream region. On the current typical CTMs, TMI solubilities are fixed as constant, which does not account for spatial (both horizontal and vertical) and temporal variations particularly found in upwind and downstream differences over East Asia (e.g., Figures S1–S4 in the Supporting Information). This study highlights the importance of TMI solubilities to determine the role of the SO_4_^2–^ formation process. Compared to the parameterization in this study, the assumptions of solubilities for dust Fe (0.45%) and Mn (5%) for the previous study conducting the modeling of Δ^17^O(SO_4_^2–^) in Beijing haze^14^ were significantly lower. This study improved SO_4_^2–^ formation in the model by implementing detailed emissions of Fe and Mn, pH-dependent rate constants for AQ(O_2_) catalyzed by TMIs, and increasing in solubilities of Fe and Mn; however, solubilities were taken as fixed parameters in time and space; hence, the detailed spatiotemporal variations of solubilities have not been accurately investigated. Thus, we propose considering spatiotemporal variations of Fe and Mn solubilities for the CTMs. To evaluate this development of CTMs and understand SO_4_^2–^ formation processes, the coordinated observation of Δ^17^O(SO_4_^2–^) and TMI solubilities along trajectories of long-range-transported air mass from upwind to downstream in East Asia will be one significant approach. These accurately modeled SO_4_^2–^ formation processes over East Asia are necessary to build SO_2_ emission regulation strategies along with carbon neutrality^60^ because the unknown response to SO_2_ emission reduction has already been reported in Western countries.^27,28^

The changes in SO_4_^2–^ oxidation processes also alter the size distribution of SO_4_^2–^ and hence direct and indirect radiative forcing,^61,62^ closely related to climate aspects. The reduction of SO_4_^2–^ in future atmospheric conditions (i.e., higher CO_2_ concentration and lower SO_2_ concentration) will increase atmospheric warming compared with the current atmospheric conditions through the slow climate response.^63,64^ The reduction of SO_4_^2–^ will also reduce atmospheric acidity and alter the magnitude, distribution, and deposition mode of nutrients supplied to the ocean in the coming decades.^65^ As we verified the important role of Fe as the catalyst on the SO_4_^2–^ formation, the declined acidity in the future will relate to weakening the role of TMI-related SO_4_^2–^ formation in the downstream of dust sources over East Asia. Given that a significant emission reduction of CO_2_ combined with a well-designed emission pathway of SO_2_ is required,^66^ our findings on the role of dust and TMI solubilities and the way to improve the modeling of SO_4_^2–^ formation will contribute to better emission regulations required for air quality and climate change.

## Supporting Information Available

Setup of regional chemical transport modeling (Section S1); configuration of CMAQ chemical mechanism for SO_4_^2–^ (Section S2); description of dust inline calculation over East Asia (Section S3); discussion on TMI solubilities (Section S4); uncertainty analysis of end member for Δ^17^O(SO_4_^2–^) of O_3_ aqueous-phase oxidation (Section S5); discussion on volcanic impacts on SO_4_^2–^ concentration and Δ^17^O(SO_4_^2–^) (Section S6); Figures S1–S6 and Tables S1–S4 (PDF)
