# Supplementary material for: Role of Dust and Iron Solubility in Sulfate Formation during the Long-Range Transport in East Asia Evidenced by 17O-Excess Signatures
Source: Environ Sci Technol. 2022 Sep 15;56(19):13634–43. doi: 10.1021/acs.est.2c03574 (PMC9535864; doi:10.1021/acs.est.2c03574)
Supplement: Supplementary file 1 — es2c03574_si_001.pdf [file es2c03574_si_001.pdf]

Supporting Information

Role of dust and iron solubility in sulfate formation during long-range transport in East Asia evidenced by ^{17}O-excess signatures

Syuichi Itahashi [a,*], Shohei Hattori [b,c,d,e*], Akinori Ito [f], Yasuhiro Sadanaga [g], Naohiro Yoshida [d,h,i], Atsushi Matsuki [e]

[a] Sustainable System Research Laboratory (SSRL), Central Research Institute of Electric Power Industry (CRIEPI), Abiko, Chiba 270-1194, Japan

[b] International Center for Isotope Effects Research (ICIER), Nanjing University, Nanjing, Jiangsu Province 210023, China

[c] School of Earth Sciences and Engineering, Nanjing University, Nanjing, Jiangsu Province 210023, China

[d] Department of Chemical Science and Engineering, School of Materials and Chemical Technology, Tokyo Institute of Technology, Midori-ku, Yokohama, Kanagawa 226-8502, Japan

[e] Institute of Nature and Environment Technology, Kanazawa University, Kakuma-machi, Kanazawa, Ishikawa 920-1192, Japan

[f] Yokohama Institute for Earth Sciences, Japan Agency for Marine-Earth Science and Technology (JAMSTEC), Kanazawa-ku, Yokohama, Kanagawa 236-0001, Japan

[g] Department of Applied Chemistry, Graduate School of Engineering, Osaka Metropolitan University, Naka-ku, Sakai, Osaka 599-8531, Japan

[h] Earth-Life Science Institute, Tokyo Institute of Technology, Meguro-ku, Tokyo 152-8551, Japan

[i] National Institute of Information and Communications Technology, Koganei, Tokyo 184-8795, Japan

*Corresponding authors: Syuichi Itahashi and Shohei Hattori

Email: isyuichi@criepi.denken.or.jp (S.I.), hattori@nju.edu.cn (S.H.)

Contents of this supporting information:

Total pages 18 includes

- Sections S1 to S6
- Figures S1 to S6
- Tables S1 to S4

S1. Regional chemical transport modeling over East Asia

The regional air quality modeling was conducted with the Community Multiscale Air Quality (CMAQ) model version 5.3.1 [1]. The features of this version of CMAQ are fitted to investigate the role of dust and Fe solubility for SO_4^{2-} formation during long-range transport. This CMAQ can explicitly treat Fe and Mn concentrations as dependent variables, calculate inline dust based on the newly developed physics-based dust scheme, and track modeled SO_4^{2-} formation processes. The simulation domain covered the entirety of East Asia with a horizontal resolution of 36 km. Forty-four non-uniform layers from the surface to 50 hPa were set vertically to represent the stratosphere-to-troposphere transport process fully [2]. To drive the CMAQ model, the meteorological fields were simulated by the Weather Research and Forecasting (WRF) model version 4.1.1 [3]. The emission dataset was prepared as follows: anthropogenic emissions were from Regional Emission inventory in ASia (REAS) version 3.1 [4]; anthropogenic emissions over Russia, which are not covered by REAS, airplane, and ship emissions were from Hemispheric Transport of Air Pollution (HTAP) version 2.2 [5]; biogenic emissions were from Model of Emissions of Gases and Aerosols from Nature (MEGAN) [6]; biomass burning emissions were from the Global Fire Emissions Database (GFED) version 4.1 [7]; and emissions from 15 active volcanoes in Japan were from observational data obtained by the Japan Meteorological Agency [8]. All of these emission inventories, except HTAP, targeted 2015, whereas HTAP targeted 2010. The initial and lateral boundary conditions for chemical species were taken from the global model of the Model for Ozone and Related Chemical Tracers (MOZART) version 4 [9]. The WRF and CMAQ simulations were conducted from 1 December 2014 to 31 December 2015, with December 2014 being discarded as a spin-up period.

S2. Configuration of CMAQ chemical mechanism for SO_4^{2-} in this study

Gas and aerosol chemistry in CMAQ were modeled using SAPRC07tic [10] and aero7 [11], respectively. The thermodynamics calculation was based on the ISORROPIA 2.1 [12]. One gas-phase reaction and five aqueous-phase reactions are involved in SO_2 oxidation (i.e., SO_4^{2-} formation) in CMAQ [13]. The gas-phase reaction is SO_2 oxidation by a hydroxyl radical (OH), and the five oxidants in the aqueous-phase pathways are hydrogen peroxide (H_2O_2), ozone (O_3), O_2 catalyzed by transition metal ions (TMIs), peroxyacetic acid (PAA), and methyl hydrogen peroxide (MHP). Our previous work demonstrated the importance of TMIs-catalyzed oxidation by O_2 in winter over Japan, and the pH dependency rate constant for the synergistic relationship between Fe and Mn was introduced [14]. The reaction rate of TMIs-catalyzed oxidation by O_2 in the original CMAQ is expressed as

$$-\frac{d[S(IV)]}{dt} = \frac{k_1[Fe(III)][S(IV)] + k_2[Mn(II)][S(IV)] + k_3[Fe(III)][Mn(II)][S(IV)]}{\left(1 + 75.0[S(VI)]^{\frac{2}{3}}\right)}, \quad (1)$$

with $k_1 = 750\ M^{-1}\ s^{-1}$, $k_2 = 2600\ M^{-1}\ s^{-1}$, and $k_3 = 1.0 \times 10^{10}\ M^{-2}\ s^{-1}$ where the denominator in Eq. (1) describes the SO_4^{2-} inhibition effect [15]. The term related to the synergetic relationship between Fe and Mn on k_3 in Eq. (1) was revised to consider pH dependency as follows.

$$\begin{cases} k_3'[H^+]^{0.67}[Fe(III)][Mn(II)][S(IV)]\ (pH \geq 4.2), \\ k_3''[H^+]^{-0.74}[Fe(III)][Mn(II)][S(IV)]\ (pH < 4.2) \end{cases} \quad (2)$$

Here, $k_3' = 2.51 \times 10^{13}$ M^{-1} s^{-1} and $k_3'' = 3.72 \times 10^7$ M^{-1} s^{-1} [16]. In this TMIs-catalyzed oxidation process, as the diurnal variation, Fe(III) was assumed to be 10% of the dissolved Fe during the day and 90% at night and Mn(II) was assumed to be the same for all dissolved Mn.
In addition to this revision of TMIs-catalyzed oxidation by O_2, the aqueous-phase pathway via NO_2 was added in CMAQ. The potential role of this aqueous NO_2 oxidation was suggested from in situ observations [17] and subsequently introduced in a modeling study [18, 19]. The rate constant expression for NO_2 was introduced as follows.

$$-\frac{\mathrm{d[S(IV)]}}{\mathrm{dt}} = k\,[\mathrm{NO_2(aq)}][\mathrm{S(IV)}] \quad (3)$$

The rate constant used for NO_2 oxidation in Eq. (3) has a range spanning one order of magnitude [20, 21], and the upper rate constant was applied [21] as follows.

$$k = 1.24 \times 10^7\ \mathrm{M^{-1}\ s^{-1}}\ (\mathrm{pH} < 5.3),\ k = 1.67 \times 10^7\ \mathrm{M^{-1}\ s^{-1}}\ (\mathrm{pH} > 8.7) \quad (4)$$

Here, the rate constant was linearly interpolated for the pH range 5.3–8.7 and used fixed values below 5.3 or above 8.7. The selection of this upper rate constant was based on our previous report to increase the possible maximum of the NO_2 aqueous oxidation process and overcome the model underestimation during winter [14].
The aqueous-phase reactions are pH dependent, and the pH is determined by the explicit speciation of crustal species (e.g., Ca^{2+}, K^+, Mg^{2+}) and transition metals (e.g., Fe, Mn), which were considered in this CMAQ model [22]. These species affect aqueous-phase oxidations of S(IV) to S(VI) by altering the pH and ionic strength of the droplets, which enabled a more realistic calculation of pH in the aqueous-phase oxidation process. The comparison of CMAQ model with available observations for fine aerosol pH and cloud pH showed reasonable agreement [23]. Although other chemical transport models, such as GEOS-Chem and CAMx, treat Fe and Mn concentrations as a fraction of other $PM_{2.5}$, the CMAQ model in this study explicitly treated Fe and Mn concentrations as dependent variables in emissions and ambient pollutants. To consider TMIs oxidation more accurately, the Transition Metal Inventory-Asia (TMI-Asia) version 1.0 [24] was used for REAS version 3.1 to calculate the portion of Fe and Mn in $PM_{2.5}$ for nine sectors. The solubility of anthropogenic Fe and Mn were set at 10% and 50%, respectively, as in the original settings in CMAQ (Table 1). The emissions of Fe and Mn were treated in detail in this study based on the latest inventory of TMI-Asia. The uncertainty in the TMIs oxidation process was attributed to the solubility. This modeling setting as the standard simulation is Exp. A in this study.

S3. East Asian dust

In East Asia, mineral dust originating from the Taklimakan and Gobi Deserts, the Loess Plateau, and Inner Mongolia is a major aerosol component and affects the radiative balance, air quality, and human health [25]. The effect of neutralization by soil dust alters acid rain over East Asia [26], and this is further related to SO_4^{2-} aqueous oxidation processes, which depend on the cloud water pH conditions. Because the modulation of pH by mineral dust has been considered in Asia, the new dust scheme in CMAQ [27] was also applied in the simulation. The Noah land surface model was applied in this study, and the calculated soil moisture was multiplied by a factor of 0.1 to avoid the

suppression of dust emissions in the calculation of aeolian dust [28]. Because of the implementation of the dust scheme, metallic ions related to mineral dust affect the gas and aerosol partitioning via ISORROPIA thermodynamics [29]. The treatment of dust in the CMAQ modeling system was introduced to capture nitrogen behavior over East Asia in our previous work [30]. The solubility of dust Fe was set as 1% in Exp. B, and the solubility of dust Mn was set as 50% (Table 1). The inclusion of the dust inline-calculation is Exp. B in this study.

S4. Solubilities of TMIs

The TMIs process is the only oxidation process to lead to negative $\Delta^{17}O(SO_4^{2-})$. A literature review reported that the solubility ranges of Fe and Mn are 0.03%–54% and 1.2%–97%, respectively [31]. The solubility of anthropogenic Fe and Mn were set at 54% and 97%, respectively, for the possible maximum production through the TMIs process in Exp. C. In addition, based on the Integrated Massively Parallel Atmospheric Chemical Transport (IMPACT) global aerosol model [32], the solubility of dust Fe [33] was increased to 3% in Exp. C (Table 1). The increase in the solubility of anthropogenic Fe [34] was also suggested based on the IMPACT global aerosol model. Because fine mineral aerosols can be acidified due to air pollution, the IMPACT global aerosol model indicated the increased dust Fe solubilities near the surface over East Asia (Figs. S1-S4). Such modulation of Fe solubilities has been supported by measurement data in the downwind region [35, 36].

S5. Uncertainty of the end member for $\Delta^{17}O(SO_4^{2-})$ of O_3 aqueous-phase oxidation

The end member and uncertainty for $\Delta^{17}O$ in aqueous-phase oxidation with O_3 [AQ(O_3)] was set to 6.4‰ ± 0.3‰ according to our previous studies [37-39]. A transfer factor of 0.25 was used to estimate $\Delta^{17}O(SO_4^{2-})$ = 6.4‰ ± 0.3‰ for AQ(O_3) based on $\Delta^{17}O$ of O_3(bulk) (= 25.6‰ ± 1.3‰) [40-42].

The assumption of a transfer factor of 0.25 was based on the slope of 1/4 between $\Delta^{17}O(SO_4^{2-})$ and $\Delta^{17}O$ of O_3(bulk) measured in a laboratory experiment [43], which hypothesized that 1/4 of $\Delta^{17}O(SO_4^{2-})$ originates from O_3 and that all three oxygen atoms of O_3 are equally likely to transfer to the product SO_4^{2-}. However, it is also proposed that O atom transfer occurs only from one of the two terminal oxygen atoms of O_3 [hereafter, O_3(term)], which is expected from theoretical calculations [44]. Given that $\Delta^{17}O$ of O_3(term) is 38.4‰ ± 2.0‰ calculated from $\Delta^{17}O$ of O_3(bulk) of 25.6‰ ± 1.3‰ [37-39], $\Delta^{17}O(SO_4^{2-})$ from S(IV) + O_3 is calculated to be 9.8‰ ± 0.5 ‰. We compared the case considering $\Delta^{17}O(SO_4^{2-})$ = 9.8‰ ± 0.5‰ for AQ(O_3) with the case of $\Delta^{17}O(SO_4^{2-})$ = 6.4‰ ± 0.3‰ for AQ(O_3) (Fig. S5). Although the seasonal trends for both cases were similar, the case assuming $\Delta^{17}O(SO_4^{2-})$ = 6.4‰ ± 0.3 ‰ for AQ(O_3) in the model showed better agreement with the observations. That similar better agreement for $\Delta^{17}O(SO_4^{2-})$ = 6.4‰ ± 0.3 ‰ for AQ(O_3) was also found in our previous study [37]. Although this determination of the end member of $\Delta^{17}O(SO_4^{2-})$ for AQ(O_3) from more comprehensive laboratory experiments is needed in future studies, the assumption of $\Delta^{17}O(SO_4^{2-})$ for AQ(O_3) does not change the trends in modeled $\Delta^{17}O(SO_4^{2-})$, nor our conclusions discussed in the main manuscript.

In a recent study, $\Delta^{17}O$ of O_3(term) in Japan was determined using a multistep nitrite-coated filter-pack system as 37.6‰ ± 1.2‰ with 1.1‰ ± 0.7‰ diurnal variation between the daytime high and the nighttime low [45]. Even when these values were used for the end-member estimate for $\Delta^{17}O(SO_4^{2-})$ for AQ(O_3) (i.e., 6.3‰ ± 0.2‰ or 9.4‰ ± 0.3‰), the conclusion was not changed substantially within the range of error.

S6. Volcanic impacts on SO_4^{2-} concentration and $\Delta^{17}O(SO_4^{2-})$

High SO_4^{2-} concentration with lower $\Delta^{17}O(SO_4^{2-})$ (Figs. 3a and 3b) was found in August. Based on the spatial distribution pattern of SO_4^{2-} (Fig. S6), this was considered to be impacted by volcanic eruptions in western Japan. In Exp. A, the low $\Delta^{17}O(SO_4^{2-})$ was due mainly to the high contribution of the gas-phase oxidation process ($\Delta^{17}O(SO_4^{2-})$ = 0‰) as discussed in the main manuscript. This low $\Delta^{17}O(SO_4^{2-})$ value was still attributed to the gas-phase oxidation process in Exp. C which enhanced the TMIs-catalyzed O_2 oxidation process by increasing TMI solubilities. The importance of TMIs-catalyzed O_2 oxidation was suggested to be the dominant process in volcanic plumes [46]; however, this study concluded the dominant role of gas-phase oxidation. The effect of volcanic eruption on SO_2 oxidation was explained by the different atmospheric chemistry between plumes in volcanoes and areas far from volcanoes.

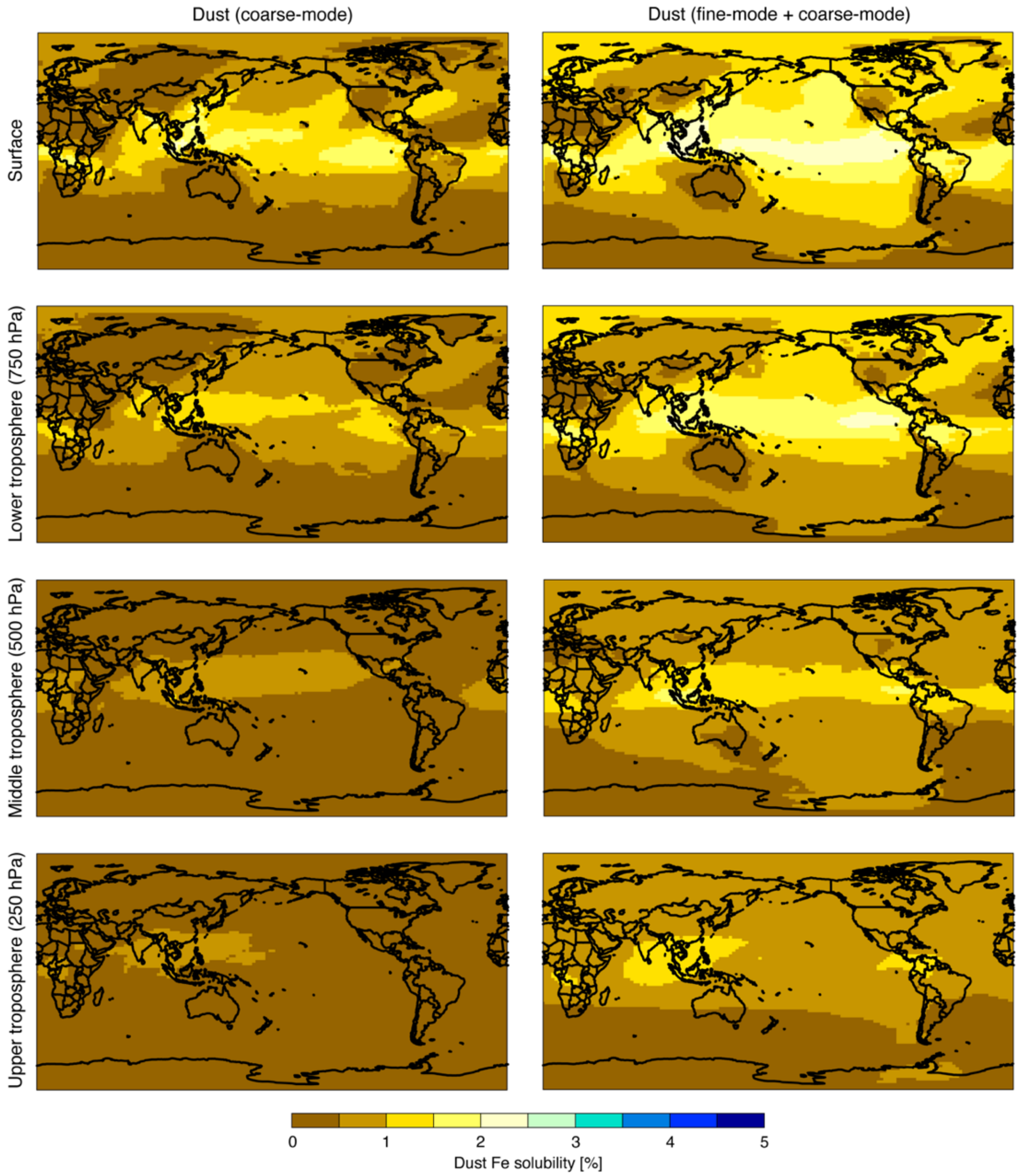

Figure S1. Annual-averaged dust Fe solubilities for (left) fine-mode and (right) total (fine-mode and coarse-mode) over the surface, lower troposphere (750 hPa), middle troposphere (500 hPa), and upper troposphere (250 hPa) simulated by the Integrated Massively Parallel Atmospheric Chemical Transport (IMPACT) global aerosol model.

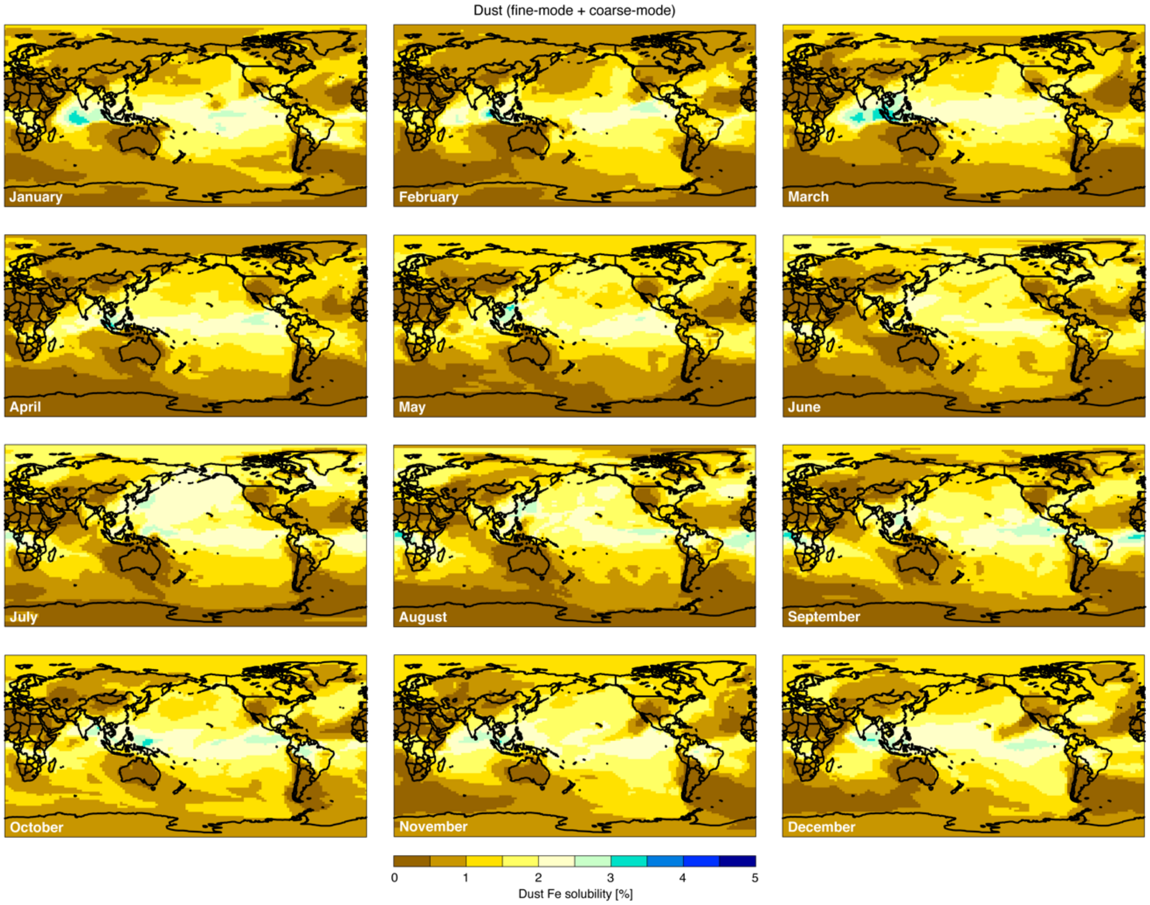

Figure S2. Monthly-averaged dust Fe solubilities for total (fine-mode and coarse-mode) over the surface simulated by the Integrated Massively Parallel Atmospheric Chemical Transport (IMPACT) global aerosol model.

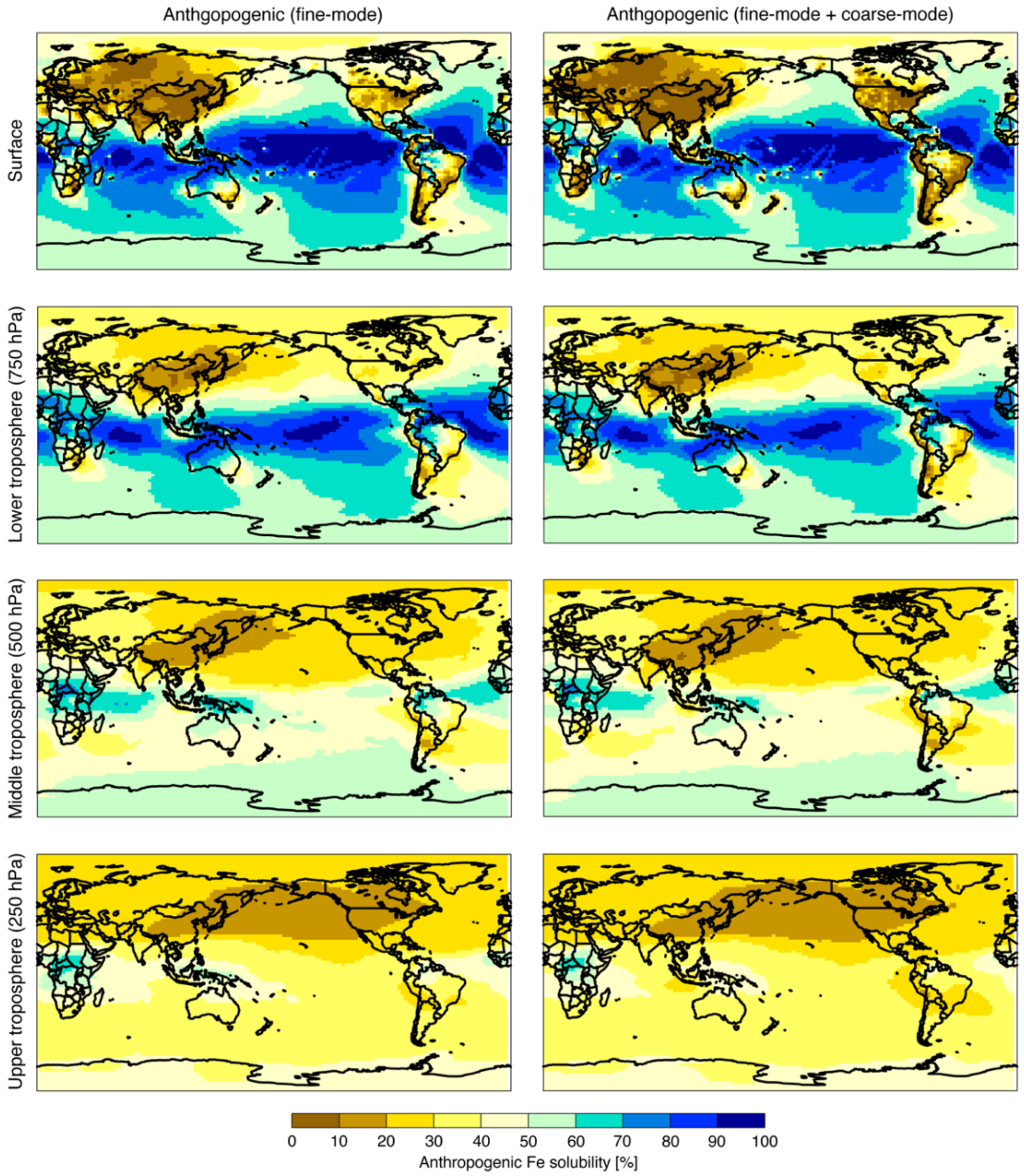

Figure S3. Annual-averaged anthropogenic Fe solubilities for (left) fine-mode and (right) total (fine-mode and coarse-mode) over the surface, lower troposphere (750 hPa), middle troposphere (500 hPa), and upper troposphere (250 hPa) simulated by the Integrated Massively Parallel Atmospheric Chemical Transport (IMPACT) global aerosol model.

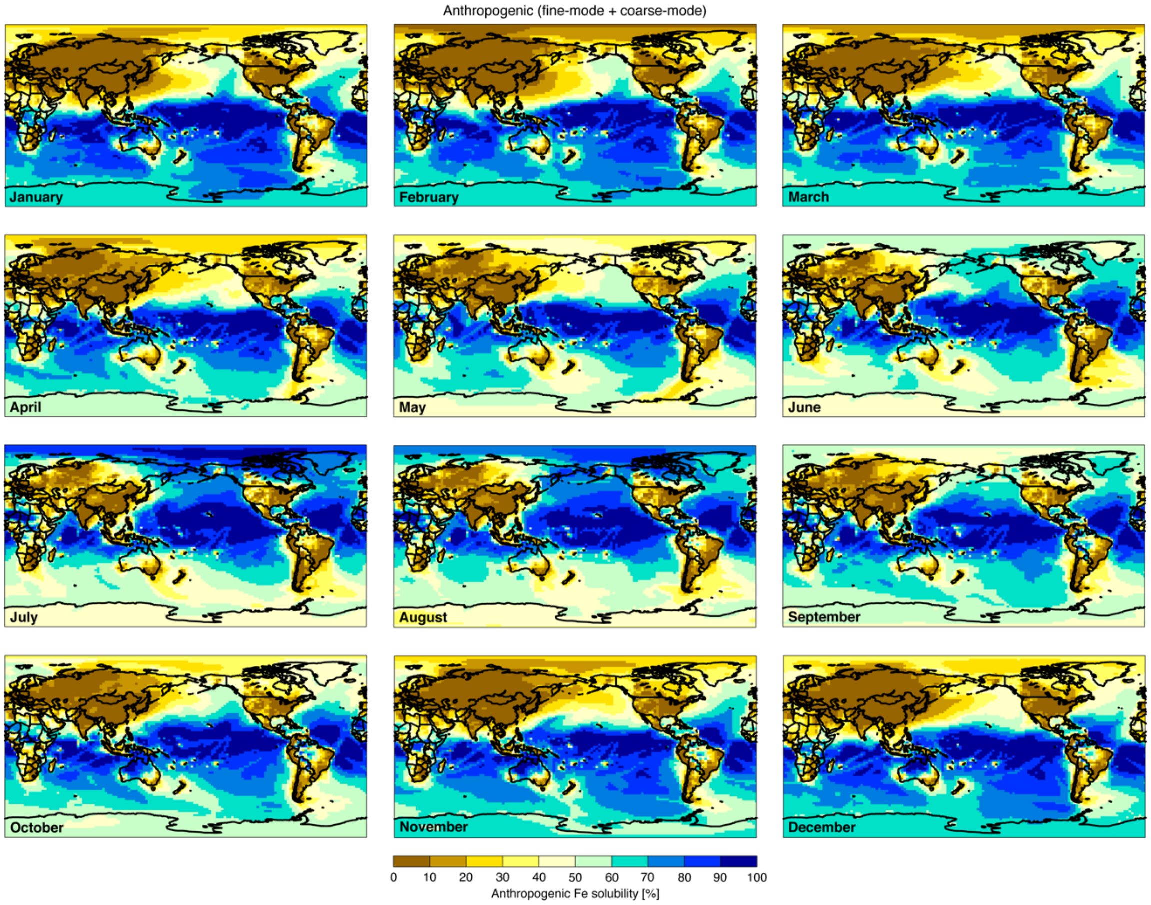

Figure S4. Monthly-averaged anthropogenic Fe solubilities for total (fine-mode and coarse-mode) over the surface simulated by the Integrated Massively Parallel Atmospheric Chemical Transport (IMPACT) global aerosol model.

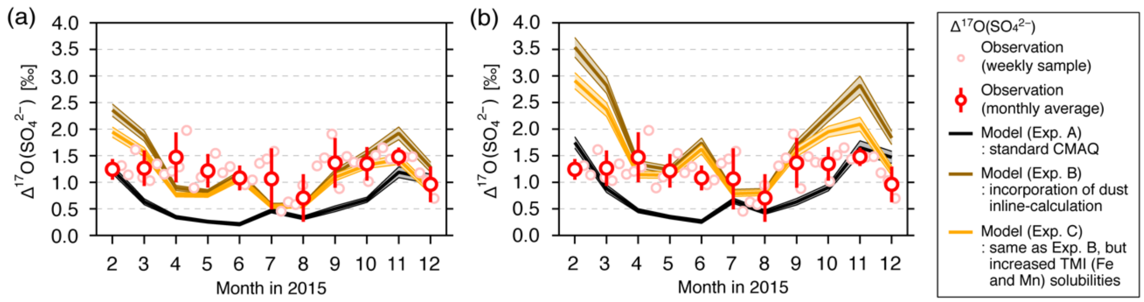

Figure S5. Observed and simulated $\Delta^{17}O(SO_4^{2-})$ from February to December in 2015 using (a) $\Delta^{17}O(SO_4^{2-})$ = 6.4‰ ± 0.3‰ for AQ(O_3) (same as Fig. 3(b) in the main manuscript but using a different *y*-axis to allow comparison with the results shown in (b)) and (b) $\Delta^{17}O(SO_4^{2-})$ = 9.8‰ ± 0.5‰ for AQ(O_3).

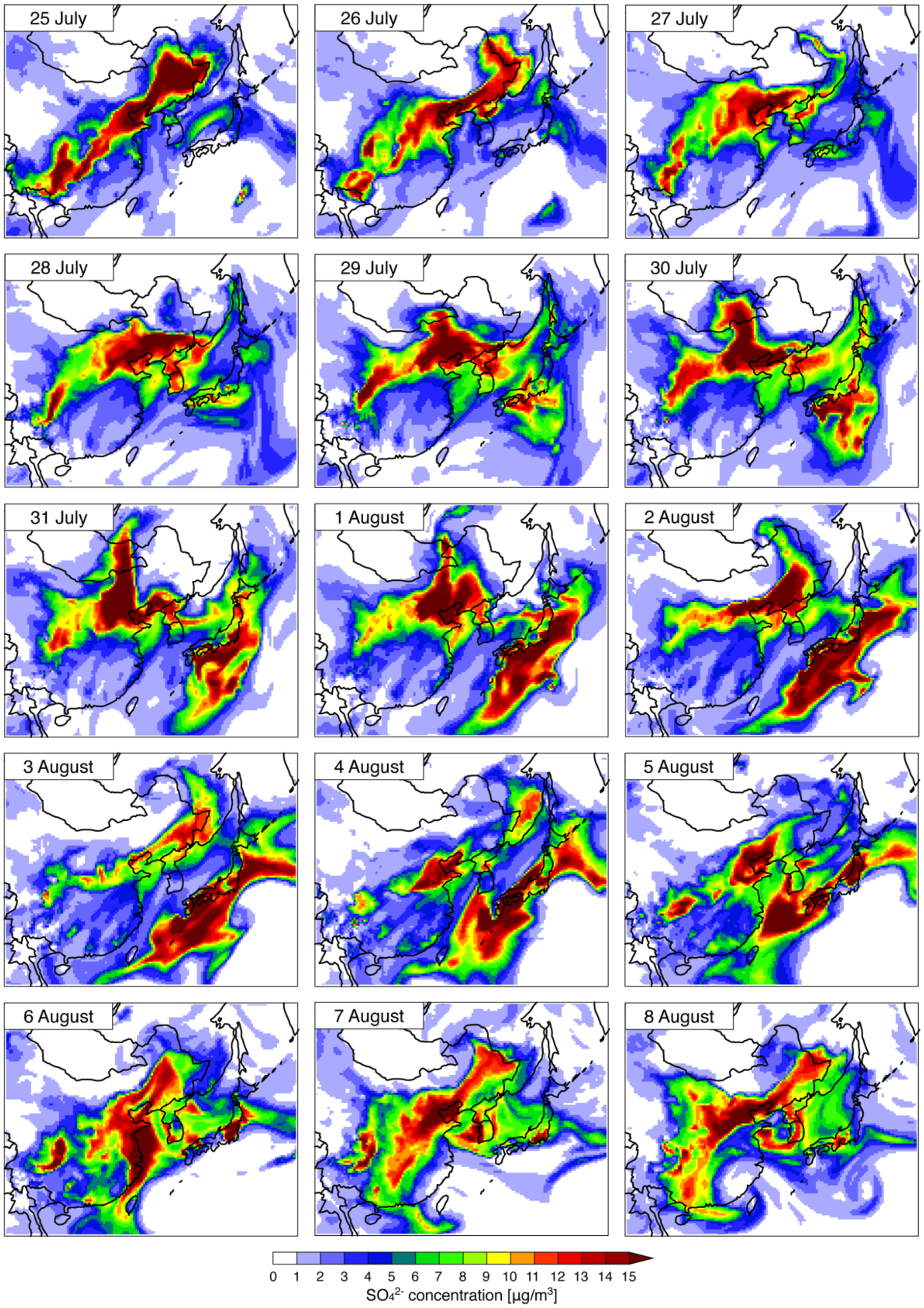

Figure S6. Daily mean of SO_4^{2-} concentration from 25 July to 8 August. The highest SO_4^{2-} concentration in Fig. 3(a) corresponds to the weekly sample between 29 July and 4 August.

Table S1. Raw observation data for SO_4^{2-} concentration and $\Delta^{17}O(SO_4^{2-})$.

Sampling date		SO_4^{2-} (μg/m^3)	$\Delta^{17}O(SO_4^{2-})$ (‰)
Start	End		
20 February	25 February	3.388	1.314
25 February	4 March	3.913	1.137
4 March	11 March	4.509	1.613
11 March	18 March	4.668	1.093
18 March	25 March	5.567	1.029
25 March	1 April	8.548	1.347
1 April	8 April	1.563	1.152
8 April	15 April	3.871	1.204
15 April	22 April	3.947	1.288
22 April	29 April	6.589	1.975
29 April	6 May	6.542	0.894
6 May	13 May	4.845	1.097
13 May	20 May	5.212	1.159
20 May	27 May	7.722	1.543
27 May	3 June	8.300	1.180
3 June	10 June	1.519	1.296
10 June	17 June	5.283	1.070
17 June	24 June	2.964	0.953
24 June	1 July	2.887	1.043
1 July	8 July	5.222	1.357
8 July	15 July	3.842	1.438
15 July	22 July	3.736	1.583
22 July	29 July	4.003	0.456
29 July	5 August	13.288	0.630
5 August	12 August	8.568	0.580
12 August	19 August	3.085	0.944
19 August	26 August	0.912	-
26 August	2 September	1.668	1.308
2 September	9 September	0.881	1.458
9 September	16 September	1.748	1.906
16 September	23 September	2.412	0.882
23 September	30 September	1.116	1.486
30 September	7 October	3.109	1.377
7 October	14 October	2.854	1.361
14 October	21 October	2.174	1.014
21 October	28 October	1.599	1.509
28 October	4 November	1.953	1.649
4 November	11 November	0.881	1.437
11 November	18 November	1.923	1.426
18 November	25 November	1.583	1.475
25 November	2 December	1.350	1.482
2 December	9 December	2.452	1.176
9 December	16 December	2.283	1.029
16 December	23 December	3.004	0.697

Note: The measurement of $\Delta^{17}O(SO_4^{2-})$ for 19 August – 26 August was a deficit.

Table S2. Observed and modeled SO_4^{2-} concentration.

	Observation	Model		
		Exp. A	Exp. B	Exp. C
February	3.58 ± 0.26	1.72	2.58	2.61
March	5.64 ± 1.71	3.38	3.61	3.66
April	4.16 ± 1.91	3.24	3.04	3.04
May	6.41 ± 1.45	5.03	4.34	4.34
June	3.51 ± 1.90	6.13	4.79	4.79
July	5.08 ± 2.86	4.20	4.13	4.13
August	4.87 ± 4.47	5.01	4.90	4.90
September	1.60 ± 0.67	2.03	1.91	1.91
October	2.35 ± 0.59	1.93	1.99	2.01
November	1.49 ± 0.41	1.63	1.77	1.87
December	2.52 ± 0.41	1.77	1.94	2.24

Note: The range of observation indicates the standard deviation when calculating the monthly average from the weekly dataset including 4% uncertainty in instruments of aerosol samples.

Table S3. Observed and modeled $\Delta^{17}O(SO_4^{2-})$.

	Observation	Model		
		Exp. A	Exp. B	Exp. C
February	1.24 ± 0.20	1.24 ± 0.08	2.36 ± 0.11	1.94 ± 0.09
March	1.26 ± 0.33	0.63 ± 0.06	1.87 ± 0.10	1.57 ± 0.08
April	1.47 ± 0.47	0.34 ± 0.03	0.89 ± 0.05	0.77 ± 0.04
May	1.21 ± 0.32	0.26 ± 0.03	0.82 ± 0.04	0.75 ± 0.04
June	1.08 ± 0.24	0.21 ± 0.03	1.15 ± 0.06	1.08 ± 0.06
July	1.06 ± 0.57	0.46 ± 0.04	0.56 ± 0.04	0.54 ± 0.04
August	0.70 ± 0.45	0.33 ± 0.03	0.57 ± 0.04	0.56 ± 0.04
September	1.36 ± 0.47	0.50 ± 0.05	1.17 ± 0.07	1.10 ± 0.07
October	1.35 ± 0.32	0.67 ± 0.05	1.53 ± 0.07	1.32 ± 0.06
November	1.48 ± 0.17	1.19 ± 0.10	1.92 ± 0.12	1.43 ± 0.09
December	0.96 ± 0.34	1.06 ± 0.09	1.28 ± 0.09	0.86 ± 0.07

Note: The range of observation indicates the standard deviation when calculating monthly weighted average from weekly dataset and including 0.11‰ uncertainty in isotopic measurements. The range of each experiment of model shows the uncertainty due to the end members of $\Delta^{17}O(SO_4^{2-})$ in oxidation pathways of 0.8‰ ± 0.2‰ for AQ(H_2O_2) and 6.4‰ ± 0.3‰ for AQ(O_3).

Table S4. Modeled oxidation process shown as a relative percentage.

	Model (Exp. A)						
	GAS	AQ(H_2O_2)	AQ(O_3)	AQ(O_2)	AQ(NO_2)	Boundary	Emission
February	45.2	16.3	14.9	4.9	3.4	13.7	1.6
March	65.8	16.8	7.3	2.9	1.0	5.4	0.8
April	77.6	10.5	3.7	0.7	1.1	5.5	0.9
May	82.6	9.2	2.6	0.4	0.6	4.1	0.5
June	75.7	18.4	2.1	0.2	0.5	2.6	0.5
July	78.0	10.5	5.6	0.1	1.2	3.4	1.1
August	76.7	14.2	3.4	0.1	1.0	3.9	0.8
September	63.7	20.8	3.9	0.3	1.9	7.8	1.6
October	56.6	14.8	6.8	2.6	2.6	15.1	1.6
November	31.3	30.4	13.6	11.3	3.5	8.1	1.8
December	32.5	26.6	12.0	13.8	3.8	9.6	1.8

	Model (Exp.B)						
	GAS	AQ(H_2O_2)	AQ(O_3)	AQ(O_2)	AQ(NO_2)	Boundary	Emission
February	23.4	4.9	34.8	7.7	10.7	8.4	10.1
March	50.2	6.1	28.0	5.2	2.6	4.9	3.0
April	68.3	4.7	13.0	2.3	3.0	5.6	3.0
May	72.8	2.3	12.2	0.7	4.0	4.4	3.7
June	56.6	6.4	21.8	1.0	5.9	2.9	5.3
July	77.3	9.2	7.3	0.2	1.3	3.5	1.2
August	75.3	10.1	8.2	0.2	1.1	4.0	1.0
September	57.4	12.4	15.3	1.0	2.9	8.2	2.8
October	44.5	4.8	21.6	5.4	5.1	14.3	4.3
November	24.3	19.5	26.6	15.7	4.1	7.3	2.6
December	27.5	22.4	16.2	19.6	3.7	8.7	1.9

	Model (Exp. C)						
	GAS	AQ(H_2O_2)	AQ(O_3)	AQ(O_2)	AQ(NO_2)	Boundary	Emission
February	22.9	4.7	28.5	15.1	10.5	8.3	10.0
March	49.3	5.8	23.5	11.2	2.5	4.8	3.0
April	68.1	4.4	11.1	4.8	3.0	5.6	3.0
May	72.8	2.2	11.2	1.8	4.0	4.4	3.7
June	56.5	6.2	20.4	2.8	5.9	2.9	5.3
July	77.2	8.9	7.0	0.9	1.3	3.5	1.2
August	75.3	9.9	7.9	0.8	1.1	4.0	1.0
September	57.1	11.9	14.3	2.8	2.8	8.1	2.8
October	43.7	4.4	18.5	9.9	5.1	14.1	4.3
November	22.0	17.0	19.5	28.8	3.4	6.9	2.5
December	22.7	18.3	10.5	36.6	2.7	7.5	1.6

References

1. Appel, K. W.; Bash, J. O.; Fahey, K. M.; Foley, K .M.; Gilliam, R. C.; Hogrefe, C.; Hutzell, W. T.; Kang, D.; Mathur, R.; Murphy, B. N.; Napelenok, S. L.; Nolte, C. G.; Pleim, J. E.; Pouliot, G. A.; Pye, H. O. T.; Ran, L.; Roselle, S. J.; Sarwar, G.; Schwede, D. B.; Sidi, F. I.; Spero, T. L.; Wong, D. C. The Community Multiscale Air Quality Model Version 5.3 and 5.3.1: System updates and evaluation. *Geosci. Model Dev.* **2021**, 14, 2867–2897.
2. Mathur, R.; Xing, J.; Gilliam, R.; Sarwar, G.; Hogrefe, C.; Pleim, J.; Pouliot, G.; Roselle, S.; Spero, T. L.; Wong, D. C.; Young, J. Extending the Community Multiscale Air Quality (CMAQ) modeling system to hemispheric scales: overview of process considerations and initial applications. *Atmos. Chem. Phys.* **2017**, 17, 12449–12474.
3. Skamarock, W. C.; Klemp, J. B.; Dudhia, J.; Gill, D. O.; Liu, Z.; Berner, J.; Wang, W.; Powers, J. G.; Duda, M. G.; Barker, D. M.; Huang, X-Y. A description of the advanced research WRF version 4. NCAR Technical Note, NCAR/TN-556+STR (NCAR, Boulder, CO, 2019), p. 162.
4. Kurokawa, J.; Ohara, T. Long-term historical trends in air pollutant emissions of in Asia: Regional Emission inventory in ASia (REAS) version 3. *Atmos. Chem. Phys.* **2020**, 20, 12761–12793.
5. Janssens-Maenhout, G.; Crippa, M.; Guizzardi, F.; Dentener, F.; Muntean, M.; Pouliot, G.; Keating, T.; Zhang, Q.; Kurokawa, J.; Wankmuller, R.; Danier van der Gon, H.; Kuenen, J.J.P.; Kilmont, Z.; Frost, G.; Darras, S.; Koffi, B.; Li, M. HTAP_v2.2: a mosaic of regional and global emission grid maps for 2008 and 2010 to study hemispheric transport of air pollution. *Atmos. Chem. Phys.* **2015**, 15, 11411–11432.
6. Guenther, A. B.; Jiang, X.; Heald, C. L.; Sakulyanontvittaya, T.; Duhl, T.; Emmons, L. K.; Wang, X. The Model of Emissions of Gases and Aerosols from Nature version 2.1 (MEGAN2.1). An extended and updated framework for modeling biogenic emissions. *Geosci. Model Dev.* **2012**, 5, 1471–1492.
7. van der Werf, G. R.; Randerson, J. T.; Giglio, L.; van Leeuwen, T. T.; Chen, Y.; Rogers, B. M.; Mu, M.; van Marle, M. J. E.; Morton, D. C.; Collatz, G. J.; Yokelson, R. J.; Kasibhatla, P. S. Global fire emissions estimates during 1997–2016. *Earth Syst. Sci. Data* **2017**, 9, 697–720.
8. Japan Meteorological Agency; http://www.data.jma.go.jp/svd/vois/data/tokyo/volcano.html [in Japanese] (Accessed 1 May, 2022).
9. Emmons, L. K.; Walters, S.; Hess, P. G.; Lamarque, J-F.; Pfister, G. G.; Fillmore, D.; Granier, C.; Guenther, A.; Kinnison, D.; Laepple, T.; Orlando, J.; Tie, X.; Tyndall, G.; Wiednmyer, C.; Baughcum, S. L.; Kloster, S. Description and evaluation of the Model for Ozone and Related chemical Tracers, version 4 (MOZART-4). *Geosci. Model Dev.* **2010**, 3, 43–67.
10. Xie, Y., Paulot, F.; Carter, W. P. L.; Nolte, C. G.; Luecken, D. J.; Hutzell, W. T.; Wennberg, P. O.; Cohen, R. C.; Pinder, R. W. Understanding the impact of recent advances in isoprene photooxidation on simulations of regional air quality. *Atmos. Chem. Phys.* **2013**, 13, 8439–8455.
11. Xu, L.; Pye, H. O. T.; He, J.; Chen, Y.; Murphy, B. N.; Ng, N. L. Experimental and model estimates of the contributions from biogenic monoterpenes and sesquiterpenes to secondary organic aerosol in the southeastern United States. *Atmos. Chem. Phys.* **2018**, 18, 12613–12637.
12. Fountoukis, C.; Nenes, A. ISORROPIA II: a computationally efficient aerosol thermodynamic equilibrium model for K^+, Ca^{2+}, Mg^{2+}, NH_4^+, Na^+, SO_4^{2-}, NO_3^-, Cl^-, H_2O aerosols. *Atmos. Chem. Phys.* **2007**, 7, 4639–4659.
13. Community Modeling and Analysis System Wiki, CMAQv5.0 sulfur chemistry; https://www.airqualitymodeling.org/index.php/CMAQv5.0_Sulfur_Chemistry (Accessed 1 March 1, 2022).
14. Itahashi, S.; Uchida, R.; Yamaji, K.; Chatani, S. Year-round modeling of sulfate aerosol over Asia through updates of aqueous-phase oxidation and gas-phase reactions with statbilized Criegee intermediates. *Atmos. Environ. X* **2021**, 12, 100123.

15. Martin, R. L.; Good, T. W. Catalyzed oxidation of sulfur dioxide in solution: The iron-manganese synergism. *Atmos. Environ.* **1991**, 25, 2395–2399.
16. Ibusuki, T.; Takeuchi, K. Sulfur dioxide oxidation by oxygen catalyzed by mixtures of manganese(II) and iron(III) in aqueous solutions at environmental reaction conditions. *Atmos. Environ.* **1987**, 21, 1555–1560.
17. Xie, Y.; Ding, A.; Nie, W.; Mao, H.; Qi, X.; Huang, X.; Xu, Z.; Kerminen, V-M.; Petäjä, T.; Chi, X.; Virkkula, A.; Boy, M.; Xue, L.; Guo, J.; Sun, J.; Yang, X.; Kulmala, M.; Fu, C. Enhanced sulfate formation by nitrogen dioxide: Implications from in situ observations at the SORPES station. *J. Geophys. Res.* **2015**, 120, 12679–12694.
18. Cheng, Y.; Zheng, G.; Wei, C.; Mu, Q.; Zheng, B.; Wang, Z.; Gao, M.; Zhang, Q.; He, K.; Carmichael, G.; Pöschl, U.; Su, H. Reactive nitrogen chemistry in aerosol water as a source of sulfate during haze evens in China. *Sci. Adv.* **2016**, 2, e1601530.
19. Wang, G.; Zhang, R.; Gomez, M.E.; Yang, L.; Zamora, M. L.; Hu, M.; Lin, Y.; Peng, J.; Guo, S.; Meng, J.; Li, J.; Cheng, C.; Hu, T.; Ren, Y.; Wang, Y.; Gao, J.; Cao, J.; An, Z.; Zhou, W.; Li, G.; Wang, J.; Tian, P.; Marrero-Ortiz, W.; Secrest, J.; Du, Z.; Zheng, J.; Shang, D.; Zeng, L.; Shao, M.; Wang, W.; Huang, Y.; Wang, Y.; Zhu, Y.; Li, Y.; Hu, J.; Pan, B.; Cai, L.; Cheng, Y.; Ji, Y.; Zhang, F.; Rosenfeld, D.; Liss, P.S.; Duce, R.A.; Kolb, C.E.; Molina, M.J. Persistent sulfate formation from London fog to Chinese haze. *Proc. Natl. Acad. Sci. U.S.A.* **2016**. 113, 13630–13635.
20. Lee, Y. N.; Schwartz, S. E. *Precipitation Scavenging, Dry Deposition and Resuspension*; Elsevier: New York, 1983; pp. 453–470.
21. Clifton, C. L.; Altstein, N.; Huie, R. E. Rate constant for the reaction of nitrogen dioxide with sulfur(IV) over the pH range 5.3–13. *Environ. Sci. Technol.* **1998**, 22, 586–589.
22. Reff, A.; Bhave, P. V.; Simon, H.; Pace, T. G.; Pouliot, G. A.; Mobley, J. D.; Houyoux, M. Emissions inventory of $PM_{2.5}$ trace elements across the United States. *Environ. Sci. Technol.* **2009**, 43, 5790–5796.
23. Pye, H. O. T.; Nenes, A.; Alexander, B.; Ault, A. P.; Barth, M. C.; Clegg, S. L.; Collett, J. L.; Fahey, K. M.; Hennigan, C. J.; Herrmann, H.; Kanakidou, M.; Kelly, J. T.; Ku, I-T.; McNeill, V. F.; Riemer, N.; Schaefer, T.; Shi, G.; Tilgner, A.; Walker, J. T.; Wang, T.; Weber, R.; Xing, J.; Zaveri, R. A.; Zuend, A. The acidity of atmospheric particles and clouds. *Atmos. Chem. Phys.* **2020**, 20, 4809–4988.
24. Kajino, M.; Hagino, H.; Fujitani, Y.; Morikawa, T.; Fukui, T.; Onishi, K.; Okuda, T.; Kajikawa, T.; Igarashi, Y. Modeling transition metals in East Asia and Japan and its emission sources. *GeoHealth* **2020**, 4, e2020GH000259.
25. Uno, I.; Eguchi, K.; Yumimoto, K.; Takemura, T.; Shimizu, A.; Uematsu, M.; Liu, Z.; Wang, Z.; Hara, Y.; Sugimoto, N. Asian dust transported one full circuit around the globe. *Nat. Geosci.* **2009**, 2, 557–560.
26. Wang, Z.; Akimoto, H.; Uno, I. Neutralization of soil aerosol and its impact on the distribution of acid rain over east Asia: Observations and model results. *J. Geophys. Res.* **2002**, 107, 4389.
27. Foroutan, H.; Young, J.; Napelenok, S. L.; Ran, L.; Appel, K. W.; Gilliam, R. C., Pleim, J. E. Development and evaluation of a physics-based windblown dust emission scheme implemented in the CMAQ modeling system. *J. Adv. Model. Earth Syst.* **2017**, 9, 585–608.
28. Darmenova, K.; Sokolik, I. N.; Shao, Y.; Marticorena, B.; Bergametti, G. Development of a physically based dust emission modeule within the Weather Research and Forecasting (WRF) model: Assessment of dust emission parameterizations and input parameters for source regions in Central and East Asia. *J. Geophys. Res.* **2009**, 114, D14201.
29. Karydis, V. A.; Tsimpodi, A. P.; Pozzer, A.; Astitha, M.; Lelieveld, J. Effects of mineral dust on global atmospheric nitrate concentrations. *Atmos. Chem. Phys.* **2016**, 16, 1491–1509.
30. Itahashi, S.; Hayashi, K.; Takeda, S.; Umezawa, Y.; Matsuda, K.; Sakurai, T.; Uno, I. Nitrogen burden from atmospheric deposition in East Asian oceans in 2010 based on high-resolution regional numerical modeling. *Environ. Poll.* **2021**, 286, 117309.

31. Shao, J.; Chen, Q.; Wang, Y.; Li, X.; He, P.; Sun, Y.; Shah, V.; Martin, R. V.; Philip, S.; Song, S.; Zhao, Y.; Xie, Z.; Zhang, L.; Alexander, B. Heterogeneous sulfate aerosol formation mechanisms during wintertime Chinese haze events: air quality model assessment using observations of sulfate oxygen isotopes in Beijing. *Atmos. Chem. Phys.* **2019**, 19, 6107–6123.
32. Ito, A.; Adebiyi, A. A.; Huang, Y.; Kok, J. F. Less atmospheric radiative heating by dust due to the synergy of coarser size and aspherical shape. *Atmos. Chem. Phys.* **2021**, 21, 16869–16891.
33. Ito, A.; Shi, Z. Delivery of anthropogenic bioavailable iron from mineral dust and combustion aerosols to the ocean. *Atmos. Chem. Phys.* **2016**, 16, 85–99.
34. Ito, A. Atmospheric processing of combustion aerosols as a source of bioavailable iron. *Environ. Sci. Technol. Lett.* **2015**, 2, 70–75.
35. Takahashi, Y.; Higashi, M.; Furukawa, T.; Mitsunobu, S. Change of iron species and iron solubility in Asian dust during the long-range transport from western China to Japan. *Atmos. Chem. Phys.* **2011**, 11, 11237–11252.
36. Sakata, K.; Kurisu, M.; Takeichi, Y.; Sakaguchi, A.; Tanimoto, H.; Tamenori, Y.; Matsuki, A.; Takahashi, Y. Iron (Fe) speciation in size-fractionated aerosol particles in the Pacific Ocean: The role of organic complexation of Fe with humic-like substances in controlling Fe solubility. *Atmos. Chem. Phys. Discuss.* **2022**, https://doi.org/10.5194/acp-2022-134.
37. Hattori, S.; Iizuka, Y.; Alexander, B.; Ishino, S.; Fujita, K.; Zhai, S.; Sherwen, T.; Oshima, N.; Uemura, R.; Yamada, A.; Suzuki, N.; Matoba, S.; Tsuruta, A.; Savarino, J.; Yoshida, N. Isotopic evidence for acidity-driven enhancement of sulfate formation after SO_2 emission control. *Sci. Adv.* **2021**, 7, eabd4610.
38. Ishino, S.; Hattori, S.; Legrand, M.; Chen, Q.; Alexander, B.; Shao, J.; Huang, J.; Jaegle, L.; Jourdain, B.; Preunkert, S.; Yamada, A.; Yoshida, N.; Savarino, J. Regional characteristics of atmospheric sulfate formation in East Antarctica imprinted on ^{17}O-excess signature. *J. Geophys. Res. Atmos.* **2021**, 126, e2020JD033583.
39. Wang, K.; Hattori, S.; Lin, M.; Ishino, S.; Alexander, B.; Kamezaki, K.; Yoshida, N.; Kang, S. Isotopic constraints on atmospheric sulfate formation pathways in the Mt. Everest region, southern Tibetan Plateau. *Atmos. Chem. Phys.* **2021**, 21, 8357–8376.
40. Vicars, W. C.; Savarino, J. Quantitative constraints on the ^{17}O-excess ($\Delta^{17}O$) signature of surface ozone: Ambient measurements from 50° N to 50° S using the nitrite-coated filter technique. *Geochim. Cosmochim. Acta* **2014**, 135, 270–287.
41. Savarino, J.; Vicars, W. C.; Legrand, M.; Preunkert, S.; Jourdain, B.; Frey, M. M.; Kukui, A.; Caillon, N.; Roca, J. G. Oxygen isotope mass balance of atmospheric nitrate at Dome C, East Antarctica, during the OPALE campaign. *Atmos. Chem. Phys.* **2016**, 16, 2659–2673.
42. Ishino, S.; Hattori, S.l Savarino, J.; Jourdain, B.; Preunnkert, S.; Legrand, M.; Caillon, N.; Barbero, A.; Kuribayashi, K.; Yoshida, N. Seasonal variations of triple oxygen isotopic compositions of atmospheric sulfate, nitrate, and ozone at Dumont d'Urville, coastal Antarctica. *Atmos. Chem. Phys.* **2017**, 17, 3713–3727.
43. Savarino, J.; Lee, C. C.; Thiemens, M. H. Laboratory oxygen isotopic study of sulfur (IV) oxidation: Origin of the mass-independent oxygen isotopic anomaly in atmospheric sulfates and sulfate mineral deposits on Earth. *J. Geophys. Res.* **2000**, 105, 29079–29088.
44. Liu, Q.; Schurter, L. M.; Muller, C. E.; Aloisio, S.; Francisco, J. S.; Margerum, D. W. Kinetics and mechanisms of aqueous ozone reactions with bromide, sulfite, hydrogen sulfite, iodide, and nitrite ions. *Inorg. Chem.* **2001**, 40, 4436–4442.
45. Xu, H.; Tsunogai, U.; Nakagawa, F.; Li, Y.; Ito, M.; Sato, K.; Tanimoto, H. Determination of the triple oxygen isotope composition of tropospheric ozone in terminal position using a multistep nitrite-coated filter-pack system. *Rapid Commun. Mass Spectrom.* **2021**, 35, e9124.
46. Galeazzo, T.; Bekki, S.; Martin, E.; Savarino, J.; Arnold, S. R. Photochemical box modeling of volcanic SO_2 oxidation: isotopic constrains. *Atmos. Chem. Phys.* **2018**, 18, 17909–17931.
